# The Effect of Folic Acid Supplementation on Endothelial Function and Arterial Stiffness Markers in Adults: A Systematic Review and Meta-Analysis

**DOI:** 10.3390/healthcare11182524

**Published:** 2023-09-13

**Authors:** Kamila Bokayeva, Małgorzata Jamka, Michalina Banaszak, Aleksandra Makarewicz-Bukowska, Ada Adamczak, Maria Chrobot, Adrianna Janicka, Natalia Jaworska, Jarosław Walkowiak

**Affiliations:** 1 Department of Pediatric Gastroenterology and Metabolic Diseases, Poznan University of Medical Sciences, Szpitalna Str. 27/33, 60-572 Poznań, Poland; kamila.bokayeva@student.ump.edu.pl (K.B.); mjamka@ump.edu.pl (M.J.); amakarewicz@ump.edu.pl (A.M.-B.); a.adamczak@ump.edu.pl (A.A.); m.chrobot@ump.edu.pl (M.C.); adawanot@wp.pl (A.J.); njaworska@ump.edu.pl (N.J.); 2 Department of Bromatology, Poznan University of Medical Sciences, Rokietnicka Str. 3, 60-806 Poznań, Poland; mi.banaszak97@gmail.com

**Keywords:** folic acid supplementation, endothelial function, arterial stiffness, flow mediated dilation, meta-analysis

## Abstract

Folic acid might improve endothelial function, but the results are inconclusive. This systematic review evaluated the effect of folic acid supplementation on endothelial parameters and arterial stiffness in adults. The study protocol was registered with the PROSPERO database (CRD42021290195). The PubMed, Web of Sciences, Cochrane and Scopus databases were searched to identify English-language randomised controlled trials of the effect of folate supplementation on arterial stiffness and endothelial function markers in adults. There were significant differences between the effect of folic acid and placebo on flow-mediated dilation (random-effects model, standardized mean differences (SMD): 0.888, 95% confidence interval (CI): 0.447, 1.329, *p* < 0.001) and monocyte chemotactic protein 1 (random-effects model, SMD: −1.364, 95% CI: −2.164, −0.563, *p* < 0.001), but there was no significant difference in the central pulse wave velocity (fixed-effects model, SMD: −0.069, 95% CI: −0.264, 0.125, *p* = 0.485) and peripheral pulse wave velocity (fixed-effects model, SMD: −0.093, 95% CI: −0.263, 0.077, *p* = 0.284). In conclusion, folic acid might have a favourable effect on endothelial function but may not affect arterial stiffness. Further studies are needed to confirm these results.

## 1. Introduction

The entire circulatory system is lined with endothelium comprising endothelial cells which play many important roles in the human body. A key function is blood flow regulation by secreting and absorbing vasoactive substances such as prostaglandins and nitric oxide (NO), which constrict and dilate blood vessels. The endothelium also controls vascular tone, filters fluids (as in the glomeruli of the kidneys), activates neutrophils in response to inflammatory mediators and participates in the restoration of vascular integrity during injury [1,2]. Endothelial cells also prevent thrombosis through anticoagulant and antiplatelet mechanisms. Endothelial dysfunction may lead to the reduced vasodilatation of blood vessels, a pro-inflammatory state and prothrombotic properties, thereby increasing the risk of numerous diseases, including peripheral vascular disease, stroke, heart disease, diabetes, insulin resistance, chronic kidney failure, tumour growth, metastasis, venous thrombosis and severe viral infection [3,4]. Endothelial function assessment encompasses a range of approaches, including both in vitro (markers of endothelial function, inflammation, oxidative stress and related factors such as endothelial nitric oxide synthase (eNOS), cell and vascular adhesive molecules, vascular endothelial growth factor (VEGF), monocyte chemotactic protein 1 (MCP-1), etc.) and in vivo methods (e.g., flow-mediated dilation (FMD) method, laser Doppler flowmetry, pulse wave velocity (PWV) measurement, pulse wave analysis) [5].

Over time, the elasticity of the walls of major arteries, notably the aorta, diminishes, leading to heightened arterial stiffness [6]. The stiffening of arterial walls is determined by common mechanisms, which include a decrease in the elastin-to-collagen ratio, the generation of elastin cross-linking, inflammation induced by reactive oxygen species, calcification, increased stiffness of vascular smooth muscle cells and endothelial dysfunction [7]. As the aorta becomes stiffer and expands due to the blood ejected from the left ventricle, it increases blood pressure. This increased aortic stiffness can also cause reflected waves from the periphery to arrive before the aortic valve closes, further straining the heart. Therefore, aortic stiffness is a significant risk factor for cardiovascular morbidity and mortality [8]. Since pulse waves travel faster in stiffer arteries, measuring pulse wave velocity is the most effective surrogate for assessing arterial stiffness in routine clinical practice [9]. 

Oxidative stress and highly reactive free radicals affect the balance of NO. In normal physiological conditions, enzymatic antioxidants such as superoxide dismutase, glutathione peroxidase, catalase and antioxidants from food (e.g., vitamin E and β-carotene and vitamin C) neutralise free radicals. However, if the amount of antioxidants is too low compared to the amount of free radicals, endothelial damage and NO imbalance can occur. Hypercholesterolemia, hyperhomocysteinemia, hypertension, diabetes, smoking, sedentary behaviour, *Chlamydia pneumonia*, *Helicobacter pylori*, *Cytomegalovirus*, *Herpes zoster virus* or *Bacteroides gingivalis* infections cause oxidative stress, thereby activating the transcription factor nuclear factor-kappa B. Subsequently, proatherogenic cytokines like tumour necrosis factor α, interleukins (IL) IL-1 and IL-6, adhesion molecules and chemokines are produced and inhibit endothelial nitric oxide synthase (eNOS) activity and, thus, NO production [10]. Excessive serum homocysteine might also contribute to oxidative damage to blood vessels, reduce NO release, cause endothelial dysfunction and decrease vasodilatation, all of which are involved in atherosclerosis [11,12].

Folate occurs naturally in foods as tetrahydrofolate [13,14] and folic acid; the fully oxidised monoglutamate form is used for fortified foods and as a dietary supplement. Folic acid is especially important for pregnant women as folic acid deficiency during pregnancy can result in growth retardation and neural tube defects (spina bifida, spinal hernia and anencephaly) in the developing foetus, low infant birth weight and preterm delivery [15]. Folate shortage reduces the cell division rate causing the production of very large red blood cells (macrocytic cells) with poorly differentiated nuclei (megaloblastic anaemia). People who abuse alcohol, with malabsorption disorders or with the non-beneficial form of methylenetetrahydrofolate reductase polymorphism, are also particularly vulnerable to folic acid deficiency.

Folic acid might enhance endothelial health through multiple ways. Folate is a cofactor in numerous biochemical reactions, including RNA and DNA synthesis or methylation. It contributes to the conversion of homocysteine to methionine and the synthesis of S-adenosyl-methionine, a methyl donor. The lack of folic acid disturbs the normal function of the methionine cycle, which increases homocysteine levels, resulting in the above-mentioned conditions. An experiment on rats specifically showed that hyperhomocysteinemia induced by folate restriction promotes arterial stiffening [16]. Thus, mild hyperhomocysteinemia and coronary artery disease (CAD) are strongly associated with low serum levels of vitamin B12 and folate [13,14].

Folic acid supplementation is also considered to improve NOS coupling, NO production and subsequent NO bioavailability [17,18,19], preventing NO imbalance and all related consequences. Additionally, it was shown that endothelium regulates arterial stiffness by releasing both NO and cytochrome-related endothelium-derived hyperpolarising factor [20,21]. With less nitric oxide to the promote relaxation and dilation of blood vessels, the arteries tend to constrict, increasing vascular resistance.

It was also suggested that folic acid may ameliorate inflammatory reactions [22,23], which can help reduce inflammation within the blood vessel walls and promote healthier endothelial function, though some studies have contradicted this statement [24,25].

Thus, folic acid might improve endothelial function [26,27], but the results are contradictory, as some studies have also shown the opposite or no effect [28,29]. Therefore, this systematic review evaluated the impact of folate acid supplementation on vascular endothelium and arterial stiffness in adults.

## 2. Materials and Methods

### 2.1. Protocol and Registration

This study was performed according to the preferred reporting items for systematic reviews and meta-analyses (PRISMA) [30] and Cochrane guidelines [31] and was registered with the International Prospective Register of Systematic Reviews (PROSPERO), registration number: CRD42021290195 [32], date of registration: 8 December 2021.

### 2.2. Information Sources and Search Strategy

The PubMed, Scopus, Web of Science and the Cochrane Library databases were searched for studies that compared the effect of folic acid supplementation with placebo on endothelial function or arterial stiffness from October 2021 to March 2023. Only randomised controlled trials were included in the analysis, and there was no restriction on publication year.

The following index terms were used:

Cochrane:

#1—(folate OR folic acid OR vitamin M OR vitamin B9 OR folacin OR folvite OR pteroylglutamic acid OR folates OR tetrahydrofolates OR formyltetrahydrofolates [Title, Abstract, Keyword]).

#2—(endothelium OR endothelial function OR endothelial dysfunction OR arterial function OR vascular function OR microvascular function OR vascular health OR vascular reactivity OR vascular stiffness OR arterial stiffness OR pulse wave analysis OR pulse wave velocity OR augmentation index OR flow-mediated dilation OR blood flow OR flow-mediated vasodilation OR adhesion molecule OR asymmetric dimethylarginine OR plasminogen activator inhibitor OR soluble vascular cell adhesion molecule OR endothelial nitric oxide synthase OR monocyte chemotactic protein OR vascular endothelial growth factor OR matrix metalloproteinase [Title, Abstract, Keyword]).

#3—#1 AND #2.

PubMed:

#1—(folate OR folic acid OR vitamin M OR vitamin B9 OR folacin OR folvite OR pteroylglutamic acid OR folates OR tetrahydrofolates OR formyltetrahydrofolates [MeSH Terms]).

#2—(endothelium OR endothelial function OR endothelial dysfunction OR arterial function OR vascular function OR microvascular function OR vascular health OR vascular reactivity OR vascular stiffness OR arterial stiffness OR pulse wave analysis OR pulse wave velocity OR augmentation index OR flow-mediated dilation OR blood flow OR flow-mediated vasodilation OR adhesion molecule OR asymmetric dimethylarginine OR plasminogen activator inhibitor OR soluble vascular cell adhesion molecule OR endothelial nitric oxide synthase OR monocyte chemotactic protein OR vascular endothelial growth factor OR matrix metalloproteinase [MeSH Terms]).

#3—#1 AND #2.

Scopus:

#1—(folate OR folic acid OR vitamin M OR vitamin B9 OR folacin OR folvite OR pteroylglutamic acid OR folates OR tetrahydrofolates OR formyltetrahydrofolates [Article title, Abstract, Keywords]).

#2—(endothelium OR endothelial function OR endothelial dysfunction OR arterial function OR vascular function OR microvascular function OR vascular health OR vascular reactivity OR vascular stiffness OR arterial stiffness OR pulse wave analysis OR pulse wave velocity OR augmentation index OR flow-mediated dilation OR blood flow OR flow-mediated vasodilation OR adhesion molecule OR asymmetric dimethylarginine OR plasminogen activator inhibitor OR soluble vascular cell adhesion molecule OR endothelial nitric oxide synthase OR monocyte chemotactic protein OR vascular endothelial growth factor OR matrix metalloproteinase [Article title, Abstract, Keywords]).

#3—#1 AND #2.

Web of Science:

#1—(folate OR folic acid OR vitamin M OR vitamin B9 OR folacin OR folvite OR pteroylglutamic acid OR folates OR tetrahydrofolates OR formyltetrahydrofolates [Topic]).

#2—(endothelium OR endothelial function OR endothelial dysfunction OR arterial function OR vascular function OR microvascular function OR vascular health OR vascular reactivity OR vascular stiffness OR arterial stiffness OR pulse wave analysis OR pulse wave velocity OR augmentation index OR flow-mediated dilation OR blood flow OR flow-mediated vasodilation OR adhesion molecule OR asymmetric dimethylarginine OR plasminogen activator inhibitor OR soluble vascular cell adhesion molecule OR endothelial nitric oxide synthase OR monocyte chemotactic protein OR vascular endothelial growth factor OR matrix metalloproteinase [Topic]).

#3—#1 AND #2.

### 2.3. Inclusion and Exclusion Criteria

The search strategy was restricted to document type (article), humans over 18 years old and studies published in English. The criteria for inclusion in the meta-analysis were intervention studies (randomised controlled trials (RCTs) parallel or crossover with data available from the first period was available) that focused on the effects of folic acid supplementation (folates, folic acid or its active form, 5-methyltetrahydrofolate orally for at least 2 weeks) on endothelial function and arterial stiffness. Participants in the control group did not receive folic acid but were allowed to use their usual medications. We considered only studies in which at least one group was administered pure folic acid while another group received a placebo. Studies in which participants were concurrently taking folate alongside other supplements but did not include groups with only folic acid supplementation were excluded. The exclusion criteria were studies conducted on animals, pregnant and lactating women, children, and non-randomised controlled trials, observational studies (cross-sectional studies, case reports, case series, ecologic studies), conference publications and abstract-only papers.

### 2.4. Data Collection Process, Extraction and Analysis

The literature search was conducted by independent reviewers based on the exclusion and inclusion criteria (MB, AM-B, AA, MC, AJ, NJ) and cross-checked by other reviewers (KB and MJ). Publications were assessed in three stages, sequentially by title, abstract and full text. Studies deemed relevant by at least one of the analysts were incorporated in the next step, and any disagreements were resolved by consensus within the review team [30]. The full text of the included articles was critically analysed, and if the full version was not available, the authors were contacted directly.

### 2.5. Data Item

The following information was extracted from each article:

1. General information: title of the article, journal name, main author and publication year;
2. Study characteristics: name and design, country (region), sample size (total number and number of subjects for each group (which included and completed the trial) and study design;
3. Type and time of intervention: studies that compared the effect of oral folic acid (form and dose) supplementation with placebo on endothelial function (the control group received a placebo or other nutrients excluding folic acid) with an intervention duration of at least two weeks;
4. Study population characteristics: age (≥18 years old), sex (% of women), body mass index (BMI (kg/m^2^)) and health condition;
5. Outcomes measured:
5.1. Main outcome:

- Endothelial function: flow-mediated dilation (FMD (%)).

5.2. Endothelial function: flow-mediated dilation (FMD (%)).

- Arterial stiffness parameters: pulse wave velocity (PWV (m/s)); pulse wave analysis (PWA), augmentation index (AIx (%));
- Endothelial function parameters: asymmetric dimethylarginine (ADMA (μmol/L)), endothelial nitric oxide synthase (eNOS (ng/mL)), monocyte chemotactic protein (MCP-1 (pg/mL)), matrix metalloproteinase (MMP), plasminogen activator inhibitor-1 (PAI-1 (AU/mL)), soluble vascular cell adhesion molecule-1 (sVCAM-1 (ng/mL)), intercellular adhesion molecule-1 (ICAM-1 (ng/mL)), vascular endothelial growth factor (VEGF (pg/mL)).

The systematic review presented the results for the parameters for which at least two papers assessing the effect of folic acid supplementation were identified [30].

### 2.6. Risk of Bias of Individual Studies

The risk of bias was assessed by three authors (KB, MJ and AJ) using the Cochrane Collaboration’s tool for randomised trials [33,34] including the following domains: bias due to randomisation, bias due to deviations from the intended intervention, bias due to missing data, bias due to outcome measurement and bias due to the selection of reported results. Criteria for low risk, some concerns and high risk of bias per the Cochrane Handbook for Systematic Reviews of Interventions were used [31]. The *robvis* (Risk-Of-Bias VISualization) was used to generate risk-of-bias plots [35].

### 2.7. Statistical Analysis

The meta-analysis was performed using Comprehensive Meta-Analysis software, version 3.0 (Biostat, Inc., Englewood, CO, USA), and a *p*-value < 0.05 was considered statistically significant. If data were presented only in a figure, the GetData Graph Digitizer 2.26.0.20 (S. Fedorov, Russia) software was used to extract the data. Post-intervention means and standard deviations (SD) were used to perform the meta-analysis. If the data had a different format, where possible, results were also summarised by entering the means and SDs as continuous outcomes to allow comparison of effect sizes across studies. When a standard error was reported, the SD was calculated from the standard error of the mean by multiplying by the same constant (the square root of the sample size). If a 95% confidence interval (CI) was available, the SD for each group was obtained by dividing the width of the CI by 3.92 and then multiplying by the square root of the group sample size. If the studies included two or more intervention groups with different doses of folic acid, the groups were combined into a single group according to the formula provided in the Cochrane guidelines [31]. If logarithmic values were presented, data were transformed back to the raw scale. If the data were presented as the median and interquartile range and after contact with the first author row data were not available, the study was not included in the meta-analysis. Data synthesis was undertaken, including a calculation of effect sizes with 95% CI using fixed-effects models (if no heterogeneity was present) and random-effects models (to analyse outcomes moderate and high with heterogeneity) with inverse variance weighting. A meta-analysis was performed when at least two studies were included that analysed data for the specific outcome. If several studies included the same population, only one paper was included in the meta-analysis. Standardised mean differences (SMDs) were used as a summary statistic to allow a comparison of effect sizes across studies. The SMD was estimated from the difference between the mean outcome values between groups divided by the pooled SD of the outcome values. Forest plots were generated to illustrate the study-specific effect sizes along with a 95% CI. Sensitivity analyses were also performed by removing each study one by one and recalculating the pooled estimates. Funnel plots were generated, and Begg’s and Egger’s tests were conducted to evaluate the presence of publication bias. Heterogeneity between studies was evaluated using Cochran Q statistics; *p* < 0.1 indicates significant heterogeneity. The I**^2^** test was also used to evaluate consistency between studies in which a value <25% indicates a low risk of heterogeneity, 25–75% indicates a moderate risk of heterogeneity and >75% indicates a high risk of heterogeneity [31]. A cumulative meta-analysis and subgroup analyses were also performed. Subgroups were defined based on the intervention duration (≤4 weeks vs. >4 weeks), mean age (<60 years vs. ≥60 years), the regions in which the studies were conducted (Europe/North America/Australia vs. other regions) and exposure to mandatory food fortification with folic acid (yes or no).

## 3. Results

### 3.1. Search Result

**Figure 1:**
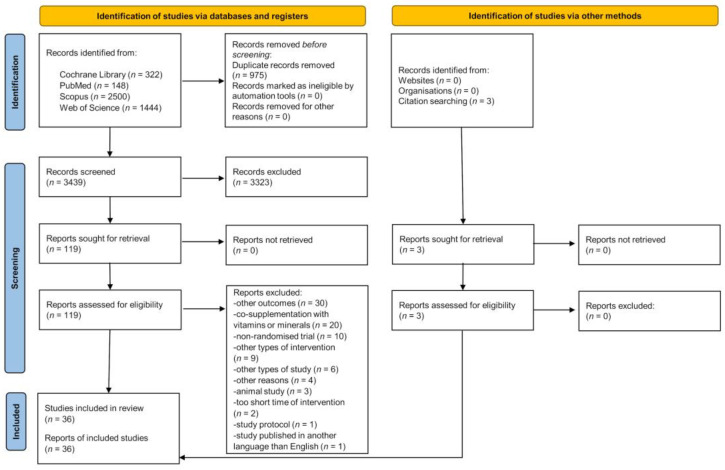
PRISMA 2020 flow diagram.

The literature search schematic is provided in Figure 1. A total of 4414 articles were identified, and 975 duplicate records were excluded. After screening the titles and abstracts, the full text of 119 papers was screened, with 36 articles deemed eligible for inclusion in this systematic review.

### 3.2. Characteristics of Included Studies

The studies were published between 1999 [36,37,38] and 2020 [39], and their characteristics are shown in Table 1. Twelve studies were performed in the United Kingdom [27,37,40,41,42,43,44,45,46,47,48,49], six in Australia [26,28,29,50,51,52], five in the Netherlands [53,54,55,56,57], three in Canada [58,59,60], three in China [38,39,61], and one each in Italy [62], Brazil [63], USA [64], Belgium [65], Turkey [66], Greece [67] and France [36].

Most studies were parallel RCTs [26,27,36,39,40,41,44,45,48,49,50,52,54,56,57,59,60,61,62,63,64,66,67] but 13 were crossover RCTs [28,29,37,38,42,43,46,47,51,53,55,58,65]. The number of participants ranged from 11 [63] to 528 [52]. Eight studies included healthy participants [29,36,37,38,47,53,55,62], but most studies involved patients with various diseases, e.g., different types of cardiovascular diseases [27,40,41,42,43,44,46,48,57,59,65,66], renal diseases [28,49,50,52], diabetes mellitus [26,54,56,58] and cognitive impairment [39,61]. Most participants were middle-aged or older [26,27,28,29,36,38,39,40,41,42,43,44,46,48,49,50,52,53,54,56,57,58,59,60,61,62,63,65,66,67], and only a few included young adults [45,47,51,55,64]. Moreover, one study did not provide information about the participants’ age [37]. Most participants were overweight or obese [26,27,29,40,41,42,43,44,45,46,48,49,50,53,54,56,57,62,66] but several studies did not report the BMI [28,36,37,38,45,47,52,58,59,61,63,65,67]. Most studies included both men and women [26,27,28,29,36,39,40,41,42,43,44,45,46,47,48,49,50,52,54,55,56,57,58,59,60,61,62,63,65,66,67], with only one study of only women [64], and four researchers did not report participant sex [37,38,51,53].

### 3.3. Characteristics of Intervention

Table 2 presents the intervention characteristics, with most studies comparing folic acid supplementation with placebo [26,28,29,37,38,42,43,44,45,46,48,49,50,51,52,53,54,55,56,57,58,62,64,65,67]. However, some studies divided the study population into three groups. Grigoletti et al. [63] compared folic acid supplementation with exercise intervention and placebo; Khandanpour et al. [27] assessed the effect of folic acid, 5-methyltetrahydrofolate and placebo; and Yilmaz et al. [66] included an additional group that received N-acetylcysteine supplementation. Pullin et al. [47] added one group that consumed foods naturally high in folate or folic acid-fortified foods, while in the study conducted by Title et al. [59], one group received multicomponent supplementation. Li et al. [39] recruited four groups supplemented with folic acid combined with docosahexaenoic acid, folic acid or docosahexaenoic acid alone and with placebo. Most studies did not detail the form of folic acid [26,28,29,36,37,38,39,40,43,44,45,48,49,50,51,53,55,60,62,63,64,65,66,67]. The remaining studies used capsules [27,57,58,59] or tablets [41,42,46,47,52,54,56,61] but one study [39] supplemented with both tablets and capsules. The dose of folic acid varied from 0.4 mg [27,40,41,47] to 15 mg per day [50,52] for between 2 [55,58] and 206 weeks [52].

### 3.4. The Effect of Folic Acid Supplementation on Flow-Mediated Dilation

**Figure 2:**
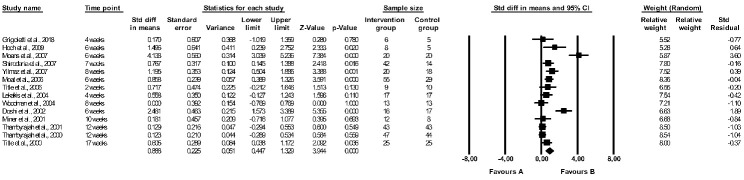
Forest plots of the effect of folic acid supplementation (favours A) vs. placebo (favours B) on flow-mediated dilation (random model) [29,40,41,44,48,49,58,59,60,63,64,65,66,67]. CI—confidence interval; Std—standard; Std diff—standard differences.

The effect of folic acid supplementation on FMD was assessed in 22 studies [29,37,38,40,41,42,43,44,46,47,48,49,53,55,58,59,60,63,64,65,66,67] (see Table 3) but only 14 papers were included in the meta-analysis [29,40,41,44,48,49,58,59,60,63,64,65,66,67]. Crossover designed RCTs without full data on the first period of intervention (before the wash-out period) were excluded [37,38,42,43,46,47,53,55]. There were significant differences between the effect of folic acid and placebo on FMD (random-effects model, SMD: 0.888, 95% CI: 0.447, 1.329, *p* < 0.001, Figure 2), with a higher FMD (more favourable effect) reported in a folic acid group compared to controls. The risk of heterogeneity among the included studies was high (Q-value = 76.029, *p* < 0.001, I^2^ = 82.901%), and a funnel plot of the standard error by standard differences in the means of FMD is shown in Figure S1. After excluding studies with a high risk of bias, the effect of folic acid and placebo on FMD remained significantly different (random-effects model, SMD: 0.899, 95% CI: 0.408, 1.391, *p* < 0.001, Figure S2). The results of sensitivity analysis are shown in Figure S3, and the results of cumulative analysis are presented in Figure S4.

### 3.5. The Effect of Folic Acid Supplementation on Pulse Wave Velocity

**Figure 3:**
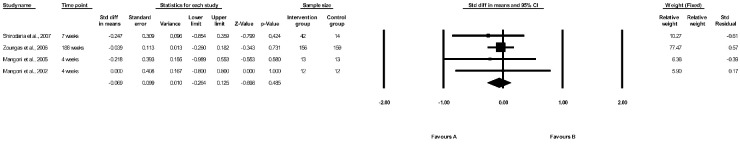
Forest plots of the effect of folic acid supplementation (favours A) vs. placebo (favours B) on central pulse wave velocity (fixed model) [26,40,45,50]. CI—confidence interval; Std—standard; Std diff—standard differences.

**Figure 4:**
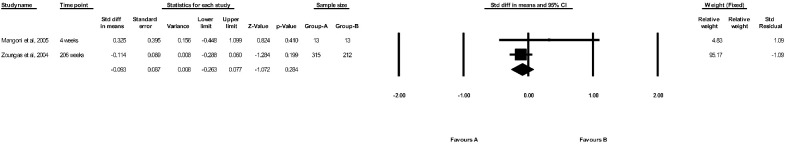
Forest plots of the effect of folic acid supplementation (favours A) vs. placebo (favours B) on peripheral pulse wave velocity (fixed model) [26,52]. CI—confidence interval; Std—standard; Std diff—standard differences.

The effect of folic acid supplementation on PWV was measured in seven studies [26,27,40,45,50,51,52] (see Table 3). Central (aortic PWV [40], aorta-femoral PWV [50,52], carotid-femoral PWV [26,45,51]) and peripheral PWV (brachial-knee PWV [27], brachial-ankle PWV [27], carotid-radial PWV [26], femoral-dorsalis PWV [51,52]) were measured. By “peripheral PWV,” we refer to methods that encompass PWV measurements of arteries located outside of the aorta, extending the assessment to more distal parts of the vascular system. Meta-analysis was performed for central PWV and peripheral PWV separately: four studies were included in the meta-analysis for the assessment of central PWV [26,40,45,50], and two studies were included for the analysis of peripheral PWV [26,52]. One study was excluded due to a crossover design without full data on the first intervention period (before the wash-out period) [51], and one study was removed due to the data presented as a median with an interquartile range [27]. The meta-analysis showed no significant difference between the effect of folic acid and placebo on central PWV (fixed-effects model, SMD: −0.069, 95% CI: −0.264, 0.125, *p* = 0.485, Figure 3). The risk of heterogeneity among the studies was low (Q-value = 0.5751, *p* = 0.9020, I^2^ = 0.000%). A funnel plot of standard error by standard differences in means of central PWV is shown in Figure S5. The results of sensitivity analysis are shown in Figure S6, and the results of cumulative analysis are presented in Figure S7. There were no differences between the effect of folic acid and placebo on peripheral PWV (fixed-effects model, SMD: −0.093, 95% CI: −0.263, 0.077, *p* = 0.284, Figure 4). The risk of heterogeneity among included studies was low (Q-value = 1.179, *p* = 0.2774, I^2^ = 15.226%). The results of sensitivity analysis are shown in Figure S8, and the results of cumulative analysis are presented in Figure S9.

### 3.6. The Effect of Folic Acid Supplementation on Monocyte Chemotactic Protein 1

**Figure 5:**
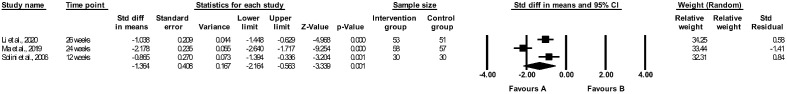
Forest plots of the effect of folic acid supplementation (favours A) vs. placebo (favours B) on monocyte chemotactic protein 1 (random-effects model) [39,61,62]. CI—confidence interval; Std—standard; Std diff—standard differences.

The effect of folic acid on MCP-1 levels was evaluated in three papers [39,61,62], and the meta-analysis revealed a significant difference between the effect of folic acid and placebo on MCP-1 (random-effects model, SMD: −1.364, 95% CI: −2.164, −0.563, *p* < 0.001, Figure 5). The placebo group showed higher levels of MCP-1 compared to the folic acid group. There was high heterogeneity between studies (Q-value = 17.814, *p* < 0.001, I^2^ = 88.773%). A funnel plot of standard error by standard differences in means of MCP-1 is shown in Figure S10. The results of sensitivity analysis are shown in Figure S11, and the results of cumulative analysis are presented in Figure S12.

### 3.7. The Effect of Folic Acid Supplementation on Other Endothelial Parameters

The effect of folic acid supplementation on other outcomes is presented in Table 3 and Table 4. Only one study assessed the effect of the intervention on Alx [52], and one study investigated the effect on the PAI-1 [36]; therefore, a meta-analysis was not possible. Two studies evaluated the effect of folic acid on sVCAM-1 concentrations [54,58] and three measured ICAM-1 levels [54,57,58] but a meta-analysis was not possible because the data were presented as the interquartile range. Out of two studies that assessed the effect of folic acid supplementation on ADMA levels [28,56], one presented data as the interquartile range [56]; thus, a meta-analysis was not performed.

### 3.8. Subgroup Analysis

The results of the subgroup analysis are presented in Figures S13–S21. Folic acid supplementation similarly enhanced FMD in studies with a short duration (≤4 weeks) and in longer-duration studies (>4 weeks) (random-effects model, SMD: 0.535, 95% CI: 0.035, 1.036, *p* = 0.036 vs. SMD: 0.996, 95% CI: 0.464, 1.528, *p* < 0.001, Figure S13). Furthermore, it demonstrated effectiveness in both younger age groups and participants aged ≥60 years (random-effects model, SMD: 1.120, 95% CI: 0.443, 1.797, *p* = 0.001 vs. SMD: 0.439, 95% CI: 0.046, 0.832, *p* = 0.029, Figure S14). Folic acid improved FMD in studies conducted in Europe, North America and Australia but did not show a significant effect in other countries (Brazil and Turkey) (random-effects model, SMD: 0.913, 95% CI: 0.423, 1.403, *p* < 0.001 vs. SMD: 0.801, 95% CI: −0.176, 1.778, *p* = 0.108, Figure S15). The absence or presence of mandatory folate fortification did not influence the effectiveness of folic acid on FMD, as positive results were observed in both cases (random-effects model, SMD: 1.043, 95% CI: 0.434, 1.652, *p* = 0.001 vs. SMD: 0.584, 95% CI: 0.202, 0.966, *p* = 0.003, Figure S16).

Subgroup analysis examining the effect of folic acid on central PWV based on the intervention duration aligns with the overall meta-analysis conducted on this parameter, revealing no significant effect regardless of the intervention duration (fixed-effects model, SMD: −0.113, 95% CI: −0.668, 0.442, *p* = 0.690 vs. SMD: −0.063, 95% CI: −0.271, 0.144, *p* = 0.551, Figure S17). Age had no impact on the effectiveness of folic acid on central PWV, as there was no improvement in any subgroup (fixed-effects model, SMD: −0.049, 95% CI: −0.254, 0.156, *p* = 0.641 vs. SMD: −0.247, 95% CI: −0.854, 0.359, *p* = 0.424, Figure S18). Neither studies with a short duration nor those with a longer duration demonstrated improvement in peripheral PWV following folic acid supplementation (fixed-effects model, SMD: 0.325, 95% CI: −0.448, 1.099, *p* = 0.410 vs. SMD: −0.114, 95% CI: −0.288, 0.060, *p* = 0.199, Figure S19). Subgroup analysis for central PWV based on countries and exposure to mandatory fortification, and for peripheral PWV based on age, countries and exposure to mandatory fortification, was not possible due to a lack of relevant studies. Folic acid supplementation successfully reduced MCP-1 levels irrespective of participants’ age (random-effects model, SMD: −0.865, 95% CI: −1.394, −0.336, *p* = 0.001 vs. SMD: −1.603, 95% CI: −2.721, −0.486, *p* = 0.005, Figure S20) or the countries where the studies were conducted (random-effects model, SMD: −0.865, 95% CI: −1.394, −0.336, *p* = 0.001 vs. SMD: −1.603, 95% CI: −2.721, −0.486, *p* = 0.005, Figure S21). Subgroup analysis for the effect of folic acid on MCP-1 levels based on intervention duration and exposure to mandatory fortification was not possible due to a lack of relevant studies.

### 3.9. Risk of Bias

**Figure 6:**
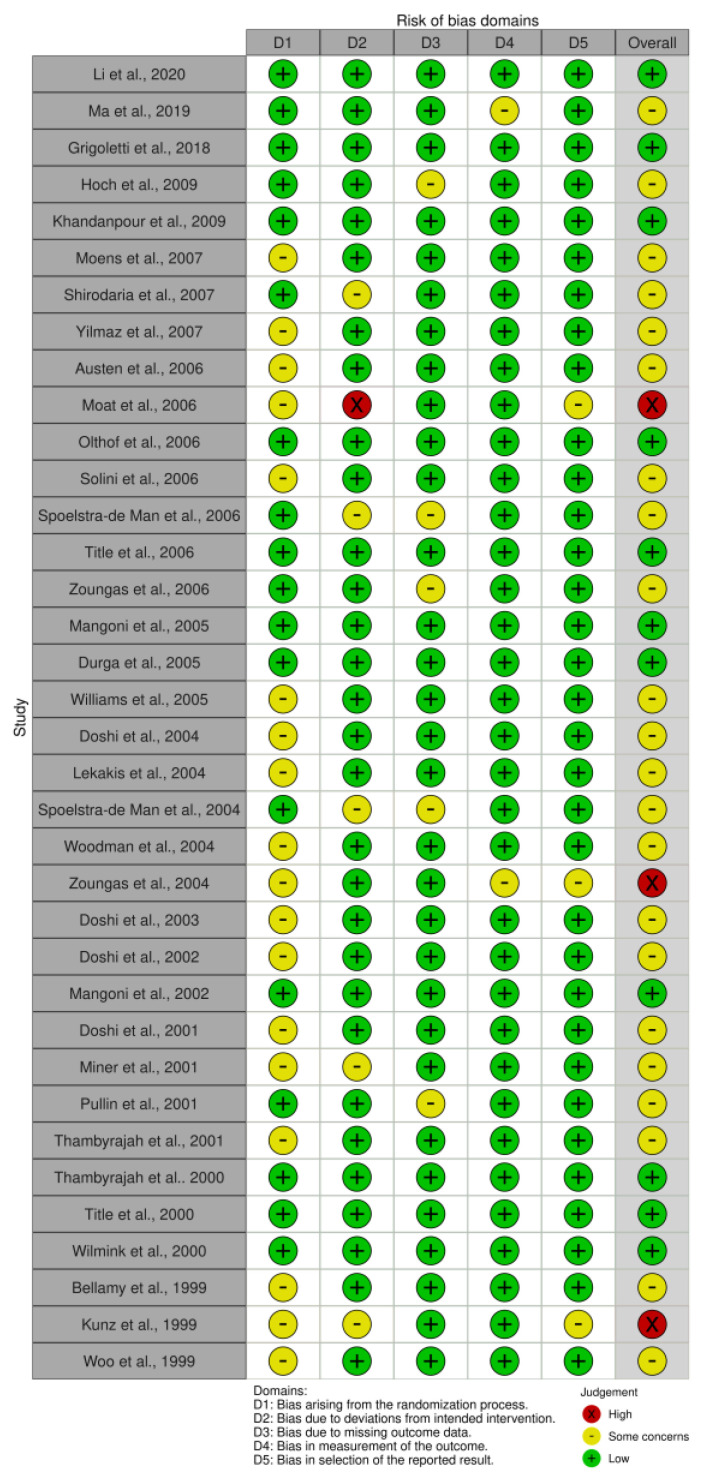
Traffic-light plot of the risk of bias [26,27,28,29,36,37,38,39,40,41,42,43,44,45,46,47,48,49,50,51,52,53,54,55,56,57,58,59,60,61,62,63,64,65,66,67].

**Figure 7:**
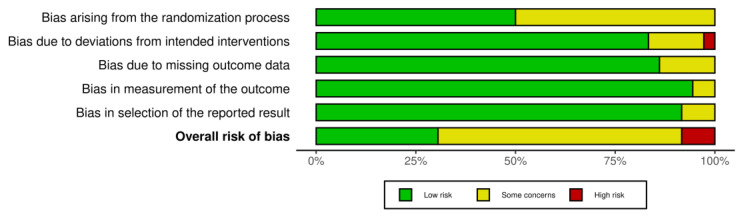
Summary plot of the risk of bias.

The results of the risk of bias assessment are presented in Figure 6 and Figure 7, with 11 studies assessed as having a low risk of bias [26,27,39,45,49,53,55,57,58,59,63], while most studies were identified to have some concerns [28,29,37,38,40,42,43,44,46,47,48,50,51,54,56,60,61,62,64,65,66,67]. Three studies were considered a high risk of bias [36,41,52]. All studies with a high risk of bias had concerns regarding the randomisation process and selection of the reported result. Additionally, two studies showed a moderate [36] and high risk [41] of bias in the domain of deviations from intended interventions, and in one study with an overall high risk of bias, there were some concerns regarding the measurement of the outcome domain [52].

## 4. Discussion

This meta-analysis suggests that folate acid supplementation has a favourable effect on the adult vascular endothelium by increasing FMD and decreasing MCP-1 levels, but do not influence arterial stiffness. Assessment of other endothelial function parameters was not performed due to an insufficient number of studies.

The mechanisms of folic acid action on the endothelium have not been fully elucidated. Reduced serum homocysteine is a known physiological effect of folate and as a cofactor in one-carbon metabolism, it promotes the remethylation of homocysteine to methionine [13,14]. Folate deficiency disturbs the methionine cycle, leading to hyperhomocysteinemia [10]. Folates may also affect endothelial function through other mechanisms, such as increasing NO bioavailability within the vascular endothelium [19]. Reduced bioavailability of tetrahydrobiopterin (BH4) causes eNOS uncoupling, which, in turn, reduces NO synthesis and increases reactive oxygen species production [68]. Shirodaria et al. [40] demonstrated that the positive effects of folic acid on endothelial function are mediated partly by improved vascular BH4 bioavailability, which leads to the restoration of uncoupled eNOS and reduced eNOS-derived superoxide production. It is also hypothesised that folic acid stimulates BH4 production from inactive oxidised dihydrobiopterin (BH2) by upregulating dihydrofolate reductase activity in the biopterin recycling pathway [69]. Moat et al. [41] showed that folic acid in vitro promoted eNOS dimerisation, suggesting that stabilisation of the NO-forming dimer may underlie the beneficial effect of folic acid on endothelial function. NO bioavailability plays a crucial role in human physiology, and the reduced ability of the endothelium to produce NO is a distinctive feature of cardiovascular diseases (CVD) [19]. Another potential folic acid protective mechanism is the upregulation of the S-adenosylmethionine to S-adenosylhomocysteine (SAM:SAH) ratio, increasing DNA methyltransferase activity and expression, altering MCP-1 and VEGF promoter methylation, and inhibiting MCP-1 and VEGF expression [70].

Our results are in line with previous meta-analyses. Notably, de Bree et al. [71] concluded that supplementation with high doses of folic acid for four weeks improved FMD. According to their analysis, folic acid dose ≤800 μg/d did not change FMD, while doses ≥5 mg/d improved it. Zamani et al. [72] also suggested that folic acid supplementation, especially in higher doses (≥5 mg/day) in cardiovascular patients, may improve endothelial function by increasing FMD and FMD% levels. Additionally, they found no significant difference in end-diastolic diameter and ICAM levels between the folic acid treatment and placebo groups. There are several differences between our study and previous meta-analyses. The first meta-analysis only assessed one parameter, %FMD, calculating the net change in FMD after folic acid supplementation compared to the placebo [71]. Moreover, the authors also included trials that combined folate supplementation with B6 and B12 vitamins, and since their article was published in 2007, our meta-analysis includes studies published after this. Zamani et al. [72] investigated the effect of folic acid supplementation on three endothelial function markers (end-diastolic diameter (EDD), FMD (%)/FMD (μm) and ICAM) in adults. Zamani et al. [72] performed separate analyses for FMD expressed in % and μm and used weighted mean differences as a summary statistic to allow comparison of effect sizes across studies. In our meta-analysis, we included both parameters in one analysis using SMD, considering it as the same marker expressed in different units. Although we used similar inclusion and exclusion criteria, our systematic review for FMD differed in the number of included studies. Our systematic review included 22 trials on FMD, but eight studies [37,38,42,43,46,47,53,55] did not proceed to meta-analysis due to their cross-over design and lack of provision of results after the first phase of the intervention (before the washout period). In their meta-analysis, Zamani et al. [72] included studies with a crossover design, and five studies [37,42,43,46,47] out of the eight excluded by us were used in their meta-analysis. Additionally, Zamani et al. [72] included in the meta-analysis three trials which we excluded according to our criteria: Woo et al. [73] presented results as a conference abstract, Hashemi et al. [74] assessed pre-eclamptic patients, and Palomba et al. study [75] had a non-randomised design. However, we included seven studies [48,49,58,60,63,64,66] which were not analysed by Zamani et al. [72] in our meta-analysis. Additionally, the results of the three other meta-analyses investigating patients with cardiovascular conditions are consistent with our findings. McRae et al. [76] concluded that supplementation with 5000–10,000 μg/d of folic acid for six weeks can increase %FMD changes and is effective in improving endothelial function in hypertensive patients. Liu et al. [77] assessed the effect of homocysteine-lowering therapy with folic acid on FMD and reported an improvement in endothelial function in CAD patients. Yi et al. [78] claimed that supplementing with 5 mg of folic acid every day for at least four weeks significantly improved FMD in CAD patients.

Several factors can potentially affect our findings. It is common to use a similar amount of folic acid supplementation regardless of sex, since the recommended dietary allowance (RDA) for folate is the same for men and women in most countries. However, Winkels et al. [79] supposed that men need more folic acid to reach folate adequacy, suggesting that the RDA for folate for men should be higher than for women because of differences in body size, but further studies are needed to confirm this. Interestingly, the meta-analysis by Asbaghi et al. [80] reported that folic acid significantly reduced serum malondialdehyde concentrations (oxidative stress marker); however, subgroup analyses found a significant effect only in females. Even though sex-differentiated research may be interesting, the possible influence of these parameters cannot be determined, so further studies are needed. Nevertheless, the authors of the trials included in our meta-analysis did not assess the results relative to sex or age. Most study participants were middle-aged or older, though some trials focused on younger populations with a wide age range across studies. Age might affect the absorption of folic acid according to studies on age-related changes in the pharmacokinetics of folic acid supplementation, with folic acid absorption being lower in middle-aged adults, while the folate rate constant of elimination increased after folic acid supplementation in young adults [81]. Subgroup analysis in our study demonstrated that age does not impact the results.

A higher BMI is associated with less supplement use, unhealthy diets and insufficient consumption of vegetables and fruits, all of which can lower folate levels [82]. Bird et al. [83] reported that obesity is associated with decreased serum folate levels and reduced folate intake and is positively associated with red blood cell folate. These findings were supported by a case-control study which showed that overweight and obese subjects had significantly lower folate intake (by 12%) and lower folate serum concentrations (by 8.5%) than the normal-weight subjects. Moreover, a significant negative association between serum folate concentrations and BMI was reported [84]. Moreover, Solini et al. [62] revealed a reduction in MCP-1 in healthy overweight volunteers (without any significant variation in BMI or fat mass), while Thambyrajah et al. [49] failed to observe an improvement in endothelial function in overweight patients with pre-dialysis chronic renal failure. Hoch et al. [64] found improved FMD in premenopausal, eumenorrheic athletic women with normal BMI. This suggests that BMI does not affect the effectiveness of folic acid treatment.

Subjects’ health status might also affect the results. Folic acid supplementation was reported to improve endothelial function in post-acute myocardial infarction [64] and CAD [40,41,44,59,66] patients. Thambyrajah et al. [48] found a greater increase in FMD from baseline in the folic acid group compared to placebo, but it was not significant in CAD patients. Cardiac transplant recipients showed no improvement in endothelial function [60]. Zoungas et al. [50] investigated the effect of high-dose folic acid on the progression of atherosclerosis and cardiovascular events in chronic renal failure patients. There was modest homocysteine lowering with no significant changes in arterial indices (PWV and AIx); therefore, folic acid therapy did not affect atheroma progression and did not improve cardiovascular morbidity or mortality. Thambyrajah et al. [49] failed to observe an improvement in endothelial function in patients with pre-dialysis chronic renal failure after high-dose folic acid supplementation.

Interestingly, Woodman et al. [29] reported no beneficial effect of folic acid on endothelial function in healthy volunteers since their endothelial function was unimpaired. In contrast, two studies [37,38] of healthy participants reported an improved FMD. Woodman et al. [29] supposed that different findings regarding the effect of folic acid on endothelial function in healthy subjects with hyperhomocysteinaemia might be explained by the absence or presence of CAD or other cardiovascular risk factors. They suggested that atherosclerotic vascular disease or additional risk may be required before the endothelial function is compromised due to hyperhomocysteinaemia, such as smoking or hyperlipidaemia [85]. Indeed, Mangoni et al. [45] found enhanced endothelial function due to folic acid supplementation in chronic smokers with no conditions affecting the cardiovascular system, though baseline homocysteine levels were in the upper normal range. Nevertheless, it might mean that folic acid supplementation does not affect endothelial function in healthy people with no cardiovascular risk factor.

Of note, different forms of folic acid were used across studies. Folate can be supplemented as folinic acid, folic acid or 5-MTHF [86]. Almost all trials in the present systematic review provided folic acid to their participants, except for three studies [27,47,63]. The first one assessed folic acid and 5-MTHF supplementation on arterial function in patients with peripheral arterial disease, showing that both treatments reduced plasma homocysteine and slightly improved brachial pressure index and brachial-knee PWV, although there was no difference in the efficacy of folic acid and 5-MTHF at the same dose [27]. The second study evaluated the effect of folinic acid supplementation compared to exercise intervention on endothelial function in HIV patients, showing that folinic acid improved endothelium-dependent vasodilatation in HIV-infected individuals with no adverse effects. Notably, aerobic exercise training possessed the same influence [63]. Finally, Pullin et al. [47] compared the effects of low folic acid supplementation, foods naturally high in folate and folic acid-fortified foods in healthy subjects, showing similar results between groups on reduced plasma homocysteine levels, with no improvement of vascular endothelial function. Taken together, the form of supplementation does not influence the meta-analysis results since only one study with folinic intervention [63] was included. Nevertheless, Scaglione et al. [86] suggested that 5-MTHF might have important advantages over synthetic folic acid and recommended this active form of folate.

Low-dose (≤800 µg) [40] and high-dose (≥2.5 mg) [37,40,41,44,58,59,60,63,64,65,66,67] folic acid beneficially affected FMD, although some studies observed no effect with high-dose supplementation [29,48,49], and one reported no effect after a low-dose intake [41]. Moat et al. [41] observed a significant improvement in FMD after 5 mg folic acid daily treatment in CAD patients, while the 400 µg dose had no effect, suggesting that folic acid enhances endothelial function in CAD in a dose-dependent manner. In contrast, Shirodaria et al. [40] found improved FMD after low-dose (400 µg) and high-dose (5 mg) folic acid supplementation, observing no additional benefit in the high-dose folic acid intervention group in a trial of CAD patients. They found that the vascular endothelium reaches its maximum capacity to take up 5-MTHF after a low-dose intake, and subsequent increases in plasma folate do not lead to a proportional rise in vascular tissue levels. Low doses of folic acid [39,61] beneficially reduced plasma concentrations of MCP-1, as did high doses (2.5 mg) [62]. PWV showed improvement after low-dose [40] and high-dose [40,45] folic acid supplementation in two studies; however, two other studies observed no effect of high-dose supplementation [50,51]. Shirodaria et al. [40] noted that changes in arterial stiffness after a high dose of folic acid were not significantly different from those after low-dose folic acid. Folic acid seems to promote endothelial function in different doses, but perhaps lower doses would have been sufficient to have an effect in studies with high doses.

At first glance, the length of the intervention does not appear to influence the effectiveness of folic acid supplementation. The shortest time for showing an improvement in FMD was two weeks [58], while the longest was 17 weeks [59]. Our subgroup analysis showed that folic acid improved FMD in studies with both short (≤4 weeks) and long (>4 weeks) intervention periods. Beneficial reduction of MCP-1 plasma concentration after folic acid therapy was reported in long-term trials of 12 weeks [62], 24 weeks [61] and 26 weeks [39]. The longest intervention of 206 weeks in chronic renal failure patients [52] succeeded in finding beneficial changes in PWV, and an intervention of 188 weeks on the same kind of patients [50] failed to find any differences between the studied groups. A much shorter trial of three weeks [51] similarly observed no improvement in PWV, while Mangoni et al. [45] observed an effect after four weeks of supplementation. Subgroup analysis indicated that neither central nor peripheral PWV is influenced by folic acid supplementation, regardless of the intervention duration.

Baseline serum folate/folic acid levels do not seem to influence the trial results. Participants with normal initial folate/folic acid mean levels showed a positive effect of folic acid treatment on FMD [40,41,44,58,59,62,63,64,66,67] and MCP-1 levels [39,62]. The same findings were observed in patients with mean baseline folate concentrations in the lower normal range [45,65]. Some studies of participants with normal initial folate levels did not find any significant changes in endothelial function [48,49] and PWV [51]. The trial conducted by Ma et al. [61] had up to 10% of folate-deficient participants in each group with a mean value of folate levels within the normal range but found a beneficial effect of folic acid on endothelial function in mild cognitive impairment.

Title et al. [59] were aware of folic acid fortification in Canada and still found evidence of improved FMD after folic acid treatment in CAD patients. Shirodaria et al. [40] supposed that the folic acid fortification programme in North America potentially impacted folic acid treatment. Given that, they recruited patients from a population without dietary folate fortification (United Kingdom). Comparing their results with others and finding no additional benefit of high-dose folic acid therapy over low-dose supplementation, they concluded that additional folate treatment in fortified populations might have no additional benefit. Hoch et al. [64] performed a study in the North American population without considering the fortification programme influence and observed a significant improvement in FMD. Further studies are needed to establish if folic treatment is beneficial for a fortified population or not. We conducted a subgroup analysis based on exposure to mandatory fortification, which indicated that mandatory folate fortification does not impact the effectiveness of folic acid on FMD. Additionally, subgroups based on countries showed that folic acid improved FMD in studies conducted in Europe, North America and Australia, but did not yield significant effects in other regions. MCP-1 levels were found to be consistent across study locations.

This meta-analysis is one of the first to investigate the effects of folic acid supplementation on endothelial function directly as well as on serum markers of endothelial dysfunction and arterial stiffness parameters. This allowed the inclusion of more studies to investigate the effects of folic acid more comprehensively and at least two weeks of supplementation advocates for increased reliability of results.

This study has several limitations. First, publication bias and a significant degree of heterogeneity across the included studies should be considered when interpreting the results. Second, regarding the crossover-designed RCTs, only the data from the first periods were included in the analysis. Since the reporting of first-period data may be dependent on statistically significant carry-over and trials reporting only paired analyses were omitted, this may lead to bias in the meta-analysis. Third, meta-regression and network meta-analysis, as well as subgroup analysis regarding sex, dosage and the health status of participants, were not performed. When conducting subgroup analyses, we observed that certain subgroups had a limited number of included studies, potentially affecting the results. Further research is needed to validate our findings. Due to the age inclusion criteria (>18 years old), the results of the meta-analysis cannot be extrapolated to a paediatric population. Among the included RCTs, interventions were performed on participants with various health conditions; for instance, FMD assessment was performed in a healthy population, HIV-infected individuals, eumenorrheic women, patients with different CVDs and type 2 diabetes mellitus, hypercholesterolaemic patients, cardiac transplant recipients and patients with chronic renal failure, making the generalisability of effects difficult. MCP-1 analysis was performed in two studies of mild cognitive impairment patients and one study of healthy volunteers with normal glucose tolerance and overweight. Moreover, the study populations differed in age so this heterogeneity should be considered.

## 5. Conclusions

Folic acid seems to have a favourable effect on FMD and MCP-1 but does not affect PWV. However, the high heterogeneity among the included studies and publication bias should be considered when interpreting the results of this meta-analysis. More well-designed RCTs are needed to assess the effect of folic acid supplementation on endothelial function and arterial stiffness markers.

## Figures and Tables

**Table 1:** Characteristics of the included studies.

Author	Year	Country (Region)	Groups	*n* Included	*n* Completed	Study Design	Studied Population	Age (Years) ^1^	BMI (kg/m^2^) ^1^	Sex [% of Women]
Li et al. [39]	2020	China (Asia)	Intervention ^2^	60	55	RCT parallel	Elderly subjects with mild cognitive impairment	70.33 ± 7.70	24.41 ± 2.47	60
			Intervention ^3^	60	53			70.20 ± 6.13	25.47 ± 3.01	60
			Intervention ^4^	60	53			71.55 ± 6.62	24.70 ± 2.52	60
			Control	60	51			70.38 ± 6.73	24.30 ± 2.93	55
Ma et al. [61]	2019	China (Asia)	Intervention ^3^	60	58	RCT parallel	Elderly subjects with mild cognitive impairment	68.42 ± 3.62	NI	63
			Intervention ^5^	60	58			69.47 ± 2.88		65
			Intervention ^6^	60	58			69.16 ± 2.46		65
			Control	60	57			68.54 ± 3.90		63
Grigoletti et al. [63]	2018	Brazil (South America)	Intervention ^3^	6	NI	RCT parallel	HIV-infected individuals, antiretroviral therapy (at least 6 months) with undetectable viral load (<50 copies/mL) and CD4 count > 200 cells/mm^3^	53 ± 3 ^8^	NI	50
			Intervention ^7^	5				52 ± 3 ^8^		40
			Control	5				48 ± 3 ^8^		60
Hoch et al. [64]	2009	USA (North America)	Intervention	NI	8	RCT parallel	Eumenorrheic woman who were not taking birth control pills and who ran at least 20 miles/week	25.0 ± 1.4 ^9^	20.7 ± 0.6 ^9^	100
			Control		5			22.4 ± 0.9 ^9^	24.4 ± 1.2 ^9^	100
Khandanpour et al. [27]	2009	United Kingdom (Europe)	Intervention ^3^	46	45	RCT parallel	Subjects with peripheral arterial disease	70.1 ± 9.0	27.5 ± 4.5	69
			Intervention ^10^	51	48			69.7 ± 7.6	27.3 ± 3.6	71
			Control	46	40			69.2 ± 8.2	26.8 ± 3.6	63
Moens et al. [65]	2007	Belgium (Europe)	Intervention	NI	20	RCT crossover	Subjects with acute myocardial infarction	57 ± 11	NI	10
			Control		20			56 ± 14		15
Shirodaria et al. [40]	2007	United Kingdom (Europe)	Intervention ^11^	56	20	RCT parallel	Subjects with coronary artery disease	62.2 ± 7.6	28.2 ± 4.0	25
			Intervention ^12^		22			62.2 ± 8.0	28 ± 3.8	9
			Control		14			64 ± 8.6	26.9 ± 4.5	14.3
Yilmaz et al. [66]	2007	Turkey (Asia)	Intervention ^3^	20	20	RCT parallel	Subjects with elevated plasma homocysteine levels (>15 μmol/L) and unequivocal angiographic evidence of coronary artery disease (>50% stenosis in one or more vessels)	52.2 ± 11.9	27.2 ± 3.8	35
			Intervention ^13^	20	16			64.8 ± 9.0	27.8 ± 3.8	15
			Control	20	18			65.5 ± 7.6	28.3 ± 4	10
Austen et al. [28]	2006	Australia (Australia)	Intervention	NI	10	RCT crossover	Renal transplant recipients	45.5 ± 11.8	NI	30
			Control							
Moat et al. [41]	2006	United Kingdom (Europe)	Intervention ^11^	NI	30	RCT parallel	Subjects with coronary artery disease	61 ± 7	28.5 ± 4.4	10
			Intervention ^12^		25			60 ± 7	29.9 ± 4.4	16
			Control		29			61 ± 7	29.6 ± 4.1	13.8
Olthof et al. [53]	2006	Netherlands (Europe)	Intervention	40	39	RCT crossover	Healthy subjects	59 ± 5	25.2 ± 2.8	NI
			Control							
Solini et al. [62]	2006	Italy (Europe)	Intervention	30	30	RCT parallel	Healthy volunteers with normal glucose tolerance and overweight	50 ± 7	27.5 ± 0.6	73.3
			Control	30	30			49 ± 8	27.4 ± 0.6	63.3
Spoelstra-de Man et al. [56] ^a^	2006	Netherlands (Europe)	Intervention	28	23	RCT parallel	Subjects with a diabetes mellitus type 2, a fasting homocysteine concentration of ≥14 μmol/L and a urinary albumin-to-creatinine ratio of at least 1 mg/mmol	64 ± 9	29 ± 4	39
			Control	23	18			66 ± 9	29 ± 3	44
Title et al. [58]	2006	Canada (North America)	Intervention	NI	19	RCT crossover	Subjects with diabetes mellitus type 2	54.5 ± 5.9	NI	52.6
			Control							
Zoungas et al. [50] ^b^	2006	Australia, New Zealand (Oceania)	Intervention	355	156	RCT parallel	Subjects with chronic renal failure of any cause	56 ± 13	26 ± 5	26.9
			Control		159			56 ± 14	27± 4	37.7
Mangoni et al. [26]	2005	Australia (Australia)	Intervention	NI	13	RCT parallel	Subjects with diabetes mellitus type 2	55.3 ± 4.3	30.5 ± 4.0	38.5
			Control		13			57.6 ± 4.7	32.3 ± 6.5	46
Williams et al. [51]	2005	Australia (Australia)	Intervention	NI	41	RCT crossover	Subjects with normal or high-normal ambulatory blood pressure (systolic: >130 to <145 mm Hg; diastolic: >80 to <90 mm Hg)	32 ± 7	24 ± 4	NI
			Control							
Durga et al. [57]	2005	Netherlands (Europe)	Intervention	530	521	RCT parallel	Men and postmenopausal women aged 50 to 70 years	60 ± 5	27 ± 3	29.9
			Control					60 ± 6	27 ± 4	26.3
Doshi et al. [42] *	2004	United Kingdom (Europe)	Intervention [31]	52	50	RCT crossover	Subjects with coronary heart disease	57 ± 8	28.5 ± 4.4	12
			Control [31]							
Lekakis et al. [67]	2004	Greece (Europe)	Intervention	NI	17	RCT parallel	Hypercholesterolaemic subjects taking statins	55.7 ± 8.3	NI	17.6
			Control		17			57.3 ± 8.8		11.8
Spoelstra-de Man et al. [54] ^a^	2004	Netherlands (Europe)	Intervention	28	23	RCT parallel	Subjects with a diabetes mellitus type 2, a fasting homocysteine concentration of ≥14 μmol/L and a urinary albumin-to-creatinine ratio of at least 1 mg/mmol	63.7 ± 8.6	29.3 ± 3.9	39
			Control	23	18			66.1 ± 8.5	28.8 ± 3.4	44
Woodman et al. [29]	2004	Australia (Australia)	Intervention	NI	26	RCT crossover	Healthy hyperhomocysteinaemic subjects	49 ± 2	28.1 ± 1.0	30.8
			Control							
Zoungas et al. [52] ^b^	2004	Australia, New Zealand (Australia)	Intervention	NI	315	RCT parallel	Subjects with chronic renal failure	58.2 ± 10.2	NI	32.4
			Control		213			56.6 ± 13.6		33.3
Doshi et al. [43] *	2003	United Kingdom (Europe)	Intervention [31]	NI	50	RCT crossover	Subjects with coronary heart disease	57 ± 8	28.5 ± 4.4	12
			Control [31]							
			Intervention [29]		33	RCT parallel	Subjects with coronary heart disease	56 ± 7	28.9 ± 5.99	9
			Control [29]							
Doshi et al. [44]	2002	United Kingdom (Europe)	Intervention	NI	16	RCT parallel	Subjects with coronary heart disease	56 ± 7	28.9 ± 5.99	9
			Control		17					
Mangoni et al. [45]	2002	United Kingdom (Europe)	Intervention	NI	12	RCT parallel	Chronic cigarette smokers	39.7 ± 3.4	25.7 ± 0.8	66.7
			Control		12			36.0 ± 3.6	24.9 ± 0.9	58.3
Doshi et al. [46]	2001	United Kingdom (Europe)	Intervention	52	50	RCT crossover	Subjects with coronary artery disease	57 ± 8	28.5 ± 4.4	12
			Control							
Miner et al. [60]	2001	Canada (North America)	Intervention ^14^	37	11	RCT parallel	Cardiac transplant recipients	55 ± 1	NI	9.1
			Intervention ^3^		12			56 ± 1		16.7
			Control		8			48 ± 1		14.3
Pullin et al. [47]	2001	United Kingdom (Europe)	Intervention ^3^	NI	42	RCT crossover	Healthy subjects	39 ± 12	NI	58
			Intervention ^15^		42					
			Control		42					
Thambyrajah et al. [48]	2001	United Kingdom (Europe)	Intervention	90	43	RCT parallel	Subjects with >50% stenosis in one or more vessels	63.0 ± 8.2	28.6 ± 4.7	14
			Control		43			63.4 ± 7.2	27.2 ± 3.5	11.6
Thambyrajah et al. [49]	2000	United Kingdom (Europe)	Intervention	50	47	RCT parallel	Subjects with chronic renal failure (serum creatinine >130 mmol/L) and a plasma homocysteine concentration >12 mmol/L	61 (57–64) ^16^	28.2 (26.6–29.8) ^16^	26
			Control	50	44			62 (59–66) ^16^	27.5 (26.1–28.8) ^16^	28
Title et al. [59]	2000	Canada (North America)	Intervention ^3^	NI	25	RCT parallel	Subjects with coronary artery disease	57.2 ± 9.8	NI	24
			Intervention ^17^		25			58.8 ± 11.6		24
			Control		25			60.6 ± 8.6		16
Wilmink et al. [55]	2000	Netherlands (Europe)	Intervention	NI	20	RCT crossover	Healthy subjects	23 ± 3.4	21.9 ± 2.7	50
			Control		20				22.8 ± 2.6	
Bellamy et al. [37]	1999	United Kingdom (Europe)	Intervention	NI	10	RCT crossover	Healthy volunteers-blood donors and members of hospital staff	NI	NI	NI
			Control		8					
Woo et al. [38]	1999	China (Asia)	Intervention	17		RCT crossover	Healthy volunteers who had no history of hypertension, diabetes mellitus, hyperlipidemia, ischemic heart disease or family history of premature atherosclerosis	54 ± 10	NI	NI
Kunz et al. [36]	1999	France (Europe)	Intervention	63	25	RCT parallel	Stable chronic haemodialysis patients who did not receive anti-epileptic drugs or other folate antagonists or oestrogens; no vitamin B12 or folate supplementation over the past 12 months	59 ± 13	NI	30
			Control		28					

^1^—mean ± standard deviation; ^2^—folic acid combined with a docosahexaenoic acid supplementation; ^3^—folic acid supplementation; ^4^—docosahexaenoic acid supplementation; ^5^—vitamin B12 supplementation; ^6^—folic acid + vitamin B12 supplementation; ^7^—exercise group; ^8^—mean ± standard error of means; ^9^—normohomocysteinemic subjects (homocysteine concentration ≤ 16 μM); ^10^—5-methyltetrahydrofolate group; ^11^—low-dose folic acid (400 μg/day); ^12^—high-dose folic acid (5 mg/day); ^13^—N-acetylcysteine group; ^14^—vitamin B6 supplementation; ^15^—group receiving foods naturally high in folate and folic acid-fortified foods; ^16^—means (95% confidence intervals); ^17^—folic acid plus antioxidants group; ^a,b^—study conducted on the same population; *—results of studies [44,46] were repeatedly presented in papers [42,43]; BMI—body mass index; NI—no information; RCT—randomised controlled trial.

**Table 2:** Characteristics of interventions.

Author	Year	Groups	Characteristic of Intervention	Form	Dose (per Day)	Time of Intervention (Weeks)
Li et al. [39]	2020	Intervention	Folic acid + docosahexaenoic acid	Tablets + capsules	800 μg + 800 mg	26
		Intervention	Folic acid		800 μg	
		Intervention	Docosahexaenoic acid		800 mg	
		Control	Placebo (corn starch + soybean oil)		NI	
Ma et al. [61]	2019	Intervention	Folic acid	Tablets	800 μg	24
		Intervention	Vitamin B12		25 µg	
		Intervention	Folic acid + vitamin B12		800 μg + 25 µg	
		Control	No intervention	NI	NI	
Grigoletti et al. [63]	2018	Intervention	Folinic acid	NI	5 mg	4
		Intervention	Aerobic exercise training		-	
		Control	Placebo		NI	
		Control	Metformin + placebo		1700 mg + NI	
Hoch et al. [64]	2009	Intervention	Folic acid	NI	10 mg	6
		Control	Placebo		NI	
Khandanpour et al. [27]	2009	Intervention	Folic acid	Capsules	400 μg	16
		Intervention	5-MTHF		400 μg	
		Control	Placebo		NI	
Moens et al. [65]	2007	Intervention	Folic acid	NI	10 mg	6
		Control	Placebo		NI	
Shirodaria et al. [40]	2007	Intervention	Folic acid	NI	400 μg	7
		Intervention	Folic acid		5 mg	
		Control	Placebo		NI	
Yilmaz et al. [66]	2007	Intervention	Folic acid	NI	5 mg	8
		Intervention	N-acetylcysteine		600 mg	
		Control	Placebo		NI	
Austen et al. [28]	2006	Intervention	Folate	NI	5 mg	14
		Control	Placebo		NI	
Moat et al. [41]	2006	Intervention	Folic acid	Tablets	400 μg	6
		Intervention	Folic acid		5 mg	
		Control	Placebo		NI	
Olthof et al. [53]	2006	Intervention	Folic acid + lactose	NI	0.8 mg + 6 g	6
		Control	Placebo (lactose)		6 g	
Solini et al. [62]	2006	Intervention	Folic acid + hypocaloric diet (1400 kcal/day, 55% carbohydrate, 25% protein and 20% fat)	NI	2.5 mg	12
		Control	Placebo + hypocaloric diet (1400 kcal/day, 55% carbohydrate, 25% protein and 20% fat)		NI	
Spoelstra-de Man et al. [54,56] ^a^	2006, 2004	Intervention	Folic acid	Tablets	5 mg	26
		Control	Placebo		NI	
Title et al. [58]	2006	Intervention	Folic acid	Capsules	10 mg	2
		Control	Placebo		NI	
Zoungas et al. [50] ^b^	2006	Intervention	Folic acid	NI	15 mg	188 ^1^
		Control	Placebo		NI	
Mangoni et al. [26]	2005	Intervention	Folic acid	NI	5 mg	4
		Control	Placebo		NI	
Williams et al. [51]	2005	Intervention	Folic acid	NI	5 mg	3
		Control	Placebo		NI	
Durga et al. [57]	2005	Intervention	Folic acid	Capsules	0.8 mg	52
		Control	Placebo		NI	
Doshi et al. [42] *	2004	Intervention	Folic acid	Tablets	5 mg	6
		Control	Placebo		NI	
Lekakis et al. [67]	2004	Intervention	Folic acid	NI	5 mg	4
		Control	Placebo		NI	
Woodman et al. [29]	2004	Intervention	Folic acid	NI	5 mg	8
		Control	Placebo		NI	
Zoungas et al. [52] ^b^	2004	Intervention	Folic acid	Tablets	15 mg	206
		Control	Placebo		NI	
Doshi et al. ^2^ [43] *	2003	Intervention	Folic acid	NI	5 mg	6
		Control	Placebo		NI	
Doshi et al. ^3^ [44]	2002	Intervention	Folic acid	NI	5 mg	6
		Control	Placebo		NI	
Mangoni et al. [45]	2002	Intervention	Folic acid	NI	5 mg	4
		Control	Placebo		NI	
Doshi et al. [46]	2001	Intervention	Folic acid	Tablets	5 mg	6
		Control	Placebo		NI	
Miner et al. [60]	2001	Intervention	Pyridoxine	NI	100 mg	10
		Intervention	Folic acid		5 mg	
		Control	Placebo		NI	
Pullin et al. [47]	2001	Intervention	Folic acid	Tablets	400 μg	17
		Intervention	Foods naturally high in folate and folic acid–fortified foods	-	~400 μg	
		Control	Placebo	NI	NI	
Thambyrajah et al. [48]	2001	Intervention	Folic acid	NI	5 mg	12
		Control	Placebo		NI	
Thambyrajah et al. [49]	2000	Intervention	Folic acid	NI	5 mg	12
		Control	Placebo		NI	
Title et al. [59]	2000	Intervention	Folic acid	Capsules	5 mg	17
		Intervention	Folic acid + vitamin C + vitamin E		5 mg + 2 g + 800 IU	
		Control	Placebo		NI	
Wilmink et al. [55]	2000	Intervention	Folic acid	NI	10 mg	2
		Control	Placebo		NI	
Bellamy et al. [37]	1999	Intervention	Folic acid	NI	5 mg	6
		Control	Placebo		NI	
Woo et al. [38]	1999	Intervention	Folic acid	NI	10 mg	4
		Control	Placebo		NI	
Kunz et al. [36]	1999	Intervention	Folic acid	NI	10 mg	8
		Control	Placebo		NI	
		Control	No intervention		NI	

^1^—patients were followed for a median of 3.6 years; ^2^—study A: 6-week high-dose folic acid; ^3^—study B: acute study with high-dose folic acid; ^a,b^—study conducted on the same population; *—results of studies [44,46] were repeatedly presented in papers [42,43]; 5-MTHF—5-methyltetrahydrofolate; NI—no information.

**Table 3:** The effect of intervention on endothelial function and arterial stiffness.

Author	Year	Group	FMD (%)			PWV (m/s)			Alx (%)		
			Pre	Post	Changes (%)	Pre	Post	Changes (%)	Pre	Post	Changes (%)
Grigoletti et al. [63]	2018	Intervention ^1^	NI	NI	7.33 ± 2.44 ^3−5^	NI	NI	NI	NI	NI	NI
		Intervention ^2^			0.04 ± 0.83 ^3−5^						
		Control			6.54 ± 0.91 ^3−5^						
Hoch et al. [64]	2009	Intervention	6.6 ± 0.8 ^3^	10.0 ± 0.9 ^3^	3.5 ± 0.6 ^3^	NI	NI	NI	NI	NI	NI
		Control	6.5 ± 0.7 ^3^	6.65 ± 0.7 ^3^	0.11 ± 0.2 ^3^						
Khandanpour et al. [27]	2009	Intervention	NI	NI	NI	10.60 (8.30–13.90) ^6,7^ 9.40 (7.70–12.50) ^6,8^	NI	−0.90 (−2.10, 0.00) ^6,7,9^ −0.50 (−1.50, 0.30) ^6,8,9^	NI	NI	NI
		Control				11.55 (9.95–15.60) ^6,7^ 9.80 (8.15–12.00) ^6,8^					
Moens et al. [65]	2007	Intervention	3.98 ± 0.35 ^3^	6.44 ± 0.56 ^3^	NI	NI	NI	NI	NI	NI	NI
		Control	4.01 ± 0.34 ^3^	4.46 ± 0.38 ^3^							
Shirodaria et al. [40]	2007	Intervention ^10^	7.71 ± 1.26 ^3,5^	11.95 ± 1.53 ^3,5^	NI	9.03 ± 1.02^3,12^	7.62 ± 0.78 ^3,12^	–1.41 ± 0.48 ^3,12^	NI	NI	NI
		Intervention ^11^	7.90 ± 1.28 ^3,5^	13.12 ± 1.7 ^3,5^		8.50 ± 0.61^3,12^	7.33 ± 0.51 ^3,12^	–1.17 ± 0.47 ^3,12^			
		Control	9.71 ± 1.02 ^3,5^	7.19 ± 1.52 ^3,5^		7.93 ± 0.71^3,12^	8.22 ± 0.90 ^3,12^	0.29 ± 0.35 ^3,12^			
Yilmaz et al. [66]	2007	Intervention ^1^	5.3 ± 2.2 ^14^	12.0 ± 6.3 ^14^	6.7 ^15^	NI	NI	NI	NI	NI	NI
		Intervention^13^	6.0 ± 2.4 ^14^	10.4 ± 3.2 ^14^	4.4 ^15^						
		Control	5.8 ± 1.9 ^14^	6.1 ± 2.7 ^14^	0.3 ^15^						
Moat et al. [41]	2006	Intervention ^10^	27.3 ± 54.5 ^14,16^	39.3 ± 31.9 ^14,16^	NI	NI	NI	NI	NI	NI	NI
		Intervention ^11^	24.4 ± 26.3 ^14,16^	99.6 ± 35.7 ^14,16^							
		Control	20.3 ± 31.0 ^14,16^	33.5 ± 21.6 ^14,16^							
Olthof et al. [53]	2006	Intervention	NI	2.8 ± 1.9 ^14^	NI	NI	NI	NI	NI	NI	NI
		Control		2.8 ± 1.8 ^14^							
Title et al. [58]	2006	Intervention	NI	5.8 ± 4.8 ^14^	NI	NI	NI	NI	NI	NI	NI
		Control	NI	3.2 ± 2.7 ^14^							
Zoungas et al. [50] ^b^	2006	Intervention	NI	NI	NI	–0.31 (–1.20–0.57) ^9,17,18^			0.1 (–5.3–5.5) ^9,18^		
		Control									
Mangoni et al. [26]	2005	Intervention	NI	NI	NI	10.1 ± 0.6 ^3,19^ 10.8 ± 0.7 ^3,20^	NI	+0.7 ± 0.6 ^3,9,19^ −0.1 ± 0.6 ^3,9,20^	NI	NI	NI
		Control				10.0 ± 0.6 ^3,19^ 10.9 ± 0.8 ^3,20^		+0.1 ± 0.4 ^3,9,19^ +0.3 ± 0.4 ^3,9,20^			
Williams et al. [51]	2005	Intervention	NI	NI	NI	7.2 ± 0.9 ^14,20^ 10.6 ± 1.5 ^14,21^	NI	–0.09 ± 0.21 ^9,14,21^ –0.10 ± 0.11 ^9,14,20^	NI	NI	NI
		Control						0.19 ± 0.25 ^9,14,21^ 0.09 ± 0.10 ^9,14,20^			
Doshi et al. [42] *	2004	Intervention	52 ± 34 ^14,16^	110 ± 43 ^14,16^	NI	NI	NI	NI	NI	NI	NI
		Control	46 ± 33 ^14,16^	47 ± 35 ^14,16^							
Lekakis et al. [67]	2004	Intervention	4.7 ± 3.2 ^14^	7.1 ± 3.1 ^14^	NI	NI	NI	NI	NI	NI	NI
		Control	5.7 ± 3.8 ^14^	5.6 ± 2.2 ^14^							
Woodman et al. [29]	2004	Intervention	7.5 ± 1.1 ^3^	8.7 ± 1.3 ^3^	+1.2 ± 1.1 ^3^	NI	NI	NI	NI	NI	NI
		Control	6.5 ± 0.7 ^3^	5.3 ± 0.7 ^3^	−1.2 ± 0.6 ^3^						
Zoungas et al. [52] ^b^	2004	Intervention	NI	NI	NI	NI	11.0 ± 3.7^14,17,22^ 10.2 ± 3.1^14,17,23^ 10.5 ± 1.8 ^14,22,24^ 10.2 ± 2.0^14,23,24^	NI	NI	21.4 ± 11.6 ^14,22^ 25.2 ± 11.5 ^14,23^	NI
		Control					9.4 ± 1.8 ^14,17,22^ 8.6 ± 1.7 ^14,17,23^ 10.8 ± 1.5 ^14,22,24^ 10.2 ± 1.6 ^14,23,24^			12.0 ± 9.3 ^14,22^ 20.4 ± 9.9 ^14,23^	
Doshi et al. [43] *^,25^	2003	Intervention	52 ± 34 ^14,16^	110 ± 43 ^14,16^	NI	NI	NI	NI	NI	NI	NI
		Control	46 ± 33 ^14,16^	47 ± 35 ^14,16^							
Doshi et al. [44] ^26^	2002	Intervention	52.5 ± 29 ^14,16^	111 ± 28 ^14,16^	NI	NI	NI	NI	NI	NI	NI
		Control	48 ± 24 ^14,16^	52 ± 19 ^14,16^							
Mangoni et al. [45]	2002	Intervention	NI	NI	NI	8.4 ± 0.3 ^3,20^	7.8 ± 0.4 ^3,20^	NI	NI	NI	NI
		Control				8.3 ± 0.5 ^3,20^	7.8 ± 0.3 ^3,20^				
Doshi et al. [46]	2001	Intervention	52 ± 34 ^14,16^	110 ± 43 ^14,16^	NI	NI	NI	NI	NI	NI	NI
		Control	46 ± 33 ^14,16^	47 ± 35 ^14,16^							
Miner et al. [60]	2001	Intervention ^27^	2.9 ± 6.7 ^14^	6.9 ± 6.3 ^14^	4.0 ± 7.6 ^14^	NI	NI	NI	NI	NI	NI
		Intervention ^1^	5.9 ± 8.3 ^14^	3.1 ± 4.8 ^14^	−5.1 ± 8.6 ^14^						
		Control	7.1 ± 5.9 ^14^	2.0 ± 7.7 ^14^	−2.8 ± 9.2 ^14^						
Pullin et al. [47]	2001	Intervention ^1^	98 ± 73 ^14,16^	114 ± 59 ^14,16^	NI	NI	NI	NI	NI	NI	NI
		Intervention ^28^		110 ± 67 ^14,16^							
		Control		118 ± 68 ^14,16^							
Thambyrajah et al. [48]	2001	Intervention	3.3 (2.2–4.3) ^18^	4.5 (3.5–5.4) ^18^	1.2 (0.7–1.8) ^18^	NI	NI	NI	NI	NI	NI
		Control	3.8 (2.6–4.9) ^18^	4.1 (3.2–5.1) ^18^	0.4 (–0.3–1.1) ^18^						
Thambyrajah et al. [49]	2000	Intervention	3.7 (2.8–4.6) ^18^	4.3 (3.5–5.2) ^18^	NI	NI	NI	NI	NI	NI	NI
		Control	2.6 (1.7–3.5) ^18^	3.9 (2.9–5.0) ^18^							
Title et al. [59]	2000	Intervention ^1^	3.2 ± 3.6 ^14^	5.2 ± 3.9 ^14^	NI	NI	NI	NI	NI	NI	NI
		Intervention ^29^	2.6 ± 2.4 ^14^	4.0 ± 3.7 ^14^							
		Control	2.7 ± 3.3 ^14^	2.9 ± 3.7 ^14^							
Wilmink et al. [55]	2000	Intervention	9.6 (7.1–12.8) ^6^	9.9 (7.5–14.1) ^6^	NI	NI	NI	NI	NI	NI	NI
		Control	10.6 (8.3–12.2) ^6^	5.8 (3.0–10.2) ^6^							
Bellamy et al. [37]	1999	Intervention	24 ± 17 ^3,30^	21 ± 14 ^3,30^	+50 ± 30 ^3,30^	NI	NI	NI	NI	NI	NI
		Control		26 ± 21 ^3,30^	+60 ± 53 ^3,30^						
Woo et al. [38]	1999	Intervention	5.7 ± 1.2 ^14^	8.2 ± 1.6 ^14^	NI	NI	NI	NI	NI	NI	NI
		Control		6.0 ± 1.3 ^14^							

^1^—folic acid only group; ^2^—exercise group; ^3^—mean ± standard error; ^4^—ml/min/100 mL; ^5^—data from figure; ^6^—median (interquartile range); ^7^—bk–PWV—brachial-knee PWV; ^8^—ba–PWV—brachial-ankle PWV; ^9^—difference between folic acid group versus placebo group; ^10^—low dose folic acid (400 μg/day); ^11^—high dose folic acid (5 mg/day); ^12^—aortic PWV; ^13^—N-acetylcysteine group; ^14^—mean ± standard deviation; ^15^—mean; ^16^—μm; ^17^—PWV (a–f)—aorto-femoral PWV; ^18^—mean (95% confidence interval); ^19^—carotid–radial PWV; ^20^—carotid–femoral PWV; ^21^—femoral-dorsal pedis arteries PWV; ^22^—male; ^23^—female; ^24^—PWV (f-d)—femoral-dorsalis PWV; ^25^—study A: 6-week high-dose folic acid; ^26^—study B: acute study with high-dose folic acid; ^27^—vitamin B6 supplementation; ^28^—group receiving foods naturally high in folate and folic acid–fortified foods; ^29^—folic acid plus antioxidants group; ^30^—mls min^−1^; ^b^—study conducted on the same population; *—results of studies [44,46] were repeatedly presented in papers [42,43]; AIx—augmentation index; FMD—flow-mediated dilatation; NI—no information; PWV—pulse wave velocity.

**Table 4:** The effect of intervention on endothelial function parameters.

Author	Year	Group	ADMA (μmol/L)			sVCAM-1 (ng/mL)			ICAM-1 (ng/mL)			MCP-1 (pg/mL)			PAI-1 (ng/L)		
			Pre	Post	Changes (%)	Pre	Post	Changes (%)	Pre	Post	Changes (%)	Pre	Post	Changes (%)	Pre	Post	Changes (%)
Li et al. [39]	2020	Intervention ^1^	NI	NI	NI	NI	NI	NI	NI	NI	NI	316.64 ± 97.65 ^4^	230.63 ± 61.81 ^4^	−86.02 ± 87.30 ^4^	NI	NI	NI
		Intervention ^2^										327.39 ± 116.91 ^4^	251.38 ± 88.90 ^4^	−76.01 ± 96.99 ^4^	NI	NI	NI
		Intervention ^3^										311.01 ± 83.49 ^4^	242.02 ± 87.39 ^4^	−68.99 ± 77.34 ^4^	NI	NI	NI
		Control										310.97 ± 115.78 ^4^	306.04 ± 82.41 ^4^	−4.94 ± 126.34 ^4^	NI	NI	NI
Ma et al. [61]	2019	Intervention ^1^	NI	NI	NI	NI	NI	NI	NI	NI	NI	802.27 ± 6.74 ^4^	783.76 ± 3.45 ^4^	NI	NI	NI	NI
		Intervention ^5^										805.99 ± 3.65 ^4^	796.32 ± 3.59 ^4^		NI	NI	NI
		Intervention ^6^										804.97 ± 6.24 ^4^	747.18 ± 3.83 ^4^		NI	NI	NI
		Control										798.77 ± 7.66 ^4^	796.44 ± 7.50 ^4^		NI	NI	NI
Austen et al. [28]	2006	Intervention	0.41 ± 0.25 ^4^	0.33 ± 0.08 ^4^	–19.8 ± 17.6 ^4^	NI	NI	NI	NI	NI	NI	NI	NI	NI	NI	NI	NI
		Control	0.58 ± 0.23 ^4^	0.48 ± 0.25 ^4^	+8.2 ± 70.3 ^4^										NI	NI	NI
Solini et al. [62]	2006	Intervention	NI	NI	NI	NI	NI	NI	NI	NI	NI	290 ± 85 ^4^	247 ± 86 ^4^	NI	NI	NI	NI
		Control										304 ± 71 ^4^	316 ± 73 ^4^		NI	NI	NI
Spoelstra-de Man et al. [56] ^a^	2006	Intervention	0.50 ± 0.08 ^4^	0.5 (0.44–0.55) ^7,8^	−0.7 ^9^	NI	NI	NI	NI	NI	NI	NI	NI	NI	NI	NI	NI
		Control	0.50 ± 0.07 ^4^	0.49 (0.42–0.56) ^7,8^	−0.2 ^9^										NI	NI	NI
Title et al. [58]	2006	Intervention	NI	NI	NI	568.5 (486.4–664.5) ^10^	557.8 (465.9–671.2) ^10^	NI	241.5 (222.5–262.2) ^10^	230.2 (212.5–249.4)^10^	NI	NI	NI	NI	NI	NI	NI
		Control				544.6 (462.2–641.6) ^10^	543.5 (457.6–645.5) ^10^		230.9 (214.4–248.6) ^10^	226.8 (212.3–242.3) ^10^					NI	NI	NI
Durga et al. [57]	2005	Intervention	NI	NI	NI	NI	NI	NI	139 (118–160) ^7^	139 (119–161) ^7^	NI	NI	NI	NI	NI	NI	NI
		Control							139 (114–165) ^7^	139 (118–170) ^7^							NI
Spoelstra-de Man et al. [54] ^a^	2004	Intervention	NI	NI	NI	1347 (1070–1640) ^7^	NI	−1 (−33–25) ^11^	668 (598–865) ^7^	NI	0 (−45–64) ^11^	NI	NI	NI	NI	NI	NI
		Control				1399 (1078–1576) ^7^		−1 (−41–79) ^11^	797 (513–1046) ^7^		2 (−29–71) ^11^				NI	NI	NI
Kunz et al. [36]	1999	Intervention	NI	NI	NI	NI	NI	NI	NI	NI	NI	NI	NI	NI	13.5 ± 13.5 ^4^	10.9 ± 4.3 ^4^	NI
		Control													14.5 ± 11.5 ^4^	17.8 ± 12.9 ^4^	NI

^1^—folic acid only group; ^2^—folic acid combined with a docosahexaenoic acid supplementation; ^3^—docosahexaenoic acid supplementation; ^4^—mean ± standard deviation; ^5^—vitamin B12 supplementation; ^6^—folic acid + vitamin B12 supplementation; ^7^—median (interquartile range); ^8^—data from figure; ^9^—mean; ^10^—geometric mean (95% confidence interval); ^11^—median (range) with 95% confidence intervals (CI); ^a^—study conducted on the same population; ADMA—asymmetric dimethylarginine; ICAM-1—intercellular adhesion molecule-1; MCP-1—monocyte chemotactic protein; NI—no information; PAI-1—plasminogen activator inhibitor type 1; sVCAM—soluble vascular cell adhesion molecule-1.

## Data Availability

Template data collection forms, data extracted from included studies, data used for analysis and any other materials used in the review are available on reasonable request from the corresponding author (J.W.).

## Supplementary Materials

The following supporting information can be downloaded at: https://www.mdpi.com/article/10.3390/healthcare11182524/s1, Figure S1: Funnel plot of standard error by standard differences in means of FMD: folic acid supplementation vs. placebo (Begg-Mazumdar test: Kendall’s tau = 0.3296, *p* = 0.1005, Egger test: bias = 3.6507 (95% confidence interval: 0.0064, 7.2951), *p* = 0.0496). Std diff—standard differences; Figure S2: Sensitivity analysis presenting mean differences with 95% confidence interval in flow-mediated dilation between folic acid supplementation (favours A) vs. placebo (favours B) (random model) after exclusion of studies with an overall high risk of bias [29,40,44,48,49,58,59,60,63,64,65,66,67]. CI—confidence interval; Std—standard; Std diff—standard differences; Figure S3: Sensitivity analysis by the jack-knife approach presenting mean differences with 95% confidence interval in flow-mediated dilation between folic acid supplementation (favours A) vs. placebo (favours B) (random model) [29,40,41,44,48,49,58,59,60,63,64,65,66,67]. CI—confidence interval; Std diff—standard differences; Figure S4: Cumulative meta-analysis of the effect of folic acid supplementation on flow-mediated dilation: (A) folic acid supplementation (favours A) vs. placebo (favours B) [29,40,41,44,48,49,58,59,60,63,64,65,66,67]. CI—confidence interval; Std—standard; Std diff—standard differences; Figure S5: Funnel plot of standard error by standard differences in means of central pulse-wave velocity: of folic acid supplementation vs. placebo (Begg-Mazumdar test: Kendall’s tau = 0.0000, *p* = 1.0000, Egger test: bias = −0.4555 (95% confidence interval: −2.1332, 1.2222), *p* = 0.3631). Std diff—standard differences; Figure S6: Sensitivity analysis by the jack-knife approach presenting mean differences with 95% confidence interval in central pulse-wave velocity between folic acid supplementation (favours A) vs. placebo (favours B) (fixed model) [26,40,45,50]. CI—confidence interval; Std diff—standard differences; Figure S7: Cumulative meta-analysis of the effect of folic acid supplementation on central pulse-wave velocity: (A) folic acid supplementation (favours A) vs. placebo (favours B) [26,40,45,60]. CI—confidence interval; Std—standard; Std diff—standard differences; Figure S8: Sensitivity analysis by the jack-knife approach presenting mean differences with 95% confidence interval in peripheral pulse-wave velocity between folic acid supplementation (favours A) vs. placebo (favours B) (fixed model) [26,52]. CI—confidence interval; Std diff—standard differences; Figure S9: Cumulative meta-analysis of the effect of folic acid supplementation on peripheral pulse-wave velocity: (A) folic acid supplementation (favours A) vs. placebo (favours B) [26,52]. CI—confidence interval; Std—standard; Std diff—standard differences; Figure S10: Funnel plot of standard error by standard differences in means of monocyte chemotactic protein: of folic acid supplementation vs. placebo (Begg-Mazumdar test: Kendall’s tau = 0.000, *p* = 1.000, Egger test: bias = 1.1897 (95% confidence interval: −297.5461, 299.9256), *p* = 0.9678). Std diff—standard differences; Figure S11: Sensitivity analysis by the jack-knife approach presenting mean differences with 95% confidence interval in monocyte chemotactic protein 1 between folic acid supplementation (favours A) vs. placebo (favours B) (random model) [39,61,62]. CI—confidence interval; Std diff—standard differences; Figure S12: Cumulative meta-analysis of the effect of folic acid supplementation on monocyte chemotactic protein: (A) folic acid supplementation (favours A) vs. placebo (favours B) [39,61,62]. CI—confidence interval; Std—standard; Std diff—standard differences; Figure S13: Subgroup meta-analysis according to intervention duration (short (≤4 weeks) vs. long (>4 weeks)) of the effect of folic acid on flow-mediated dilation: folic acid supplementation (favours A) vs. placebo (favours B) [29,40,41,44,48,49,58,59,60,63,64,65,66,67]; Figure S14: Subgroup meta-analysis according to age (<60 years vs. ≥60 years) of the effect of folic acid on flow-mediated dilation: folic acid supplementation (favours A) vs. placebo (favours B) [29,40,41,44,48,49,58,59,60,63,64,65,66,67]; Figure S15: Subgroup meta-analysis according to countries (Europe/North America/Australia vs. other regions) of the effect of folic acid on flow-mediated dilation: folic acid supplementation (favours A) vs. placebo (favours B) [29,40,41,44,48,49,58,59,60,63,64,65,66,67]; Figure S16: Subgroup meta-analysis according to exposure to mandatory folate fortification (yes vs. no) of the effect of folic acid on flow-mediated dilation: folic acid supplementation (favours A) vs. placebo (favours B) [29,40,41,44,48,49,58,59,60,63,64,65,66,67]; Figure S17: Subgroup meta-analysis according to intervention duration (short (≤4 weeks) vs. long (>4 weeks)) of the effect of folic acid on central pulse-wave velocity: folic acid supplementation (favours A) vs. placebo (favours B) [26,40,45,50]; Figure S18: Subgroup meta-analysis according to age (<60 years vs. ≥60 years) of the effect of folic acid on central pulse-wave velocity: folic acid supplementation (favours A) vs. placebo (favours B) [26,40,45,50]; Figure S19: Subgroup meta-analysis according to intervention duration (short (≤4 weeks) vs. long (>4 weeks)) of the effect of folic acid on peripheral pulse-wave velocity: folic acid supplementation (favours A) vs. placebo (favours B) [26,52]; Figure S20: Subgroup meta-analysis according to age (<60 years vs. ≥60 years) of the effect of folic acid on monocyte chemotactic protein 1: folic acid supplementation (favours A) vs. placebo (favours B) [39,61,62]; Figure S21: Subgroup meta-analysis according to countries (Europe/North America/Australia vs. other regions) of the effect of folic acid on monocyte chemotactic protein 1: folic acid supplementation (favours A) vs. placebo (favours B) [39,61,62].
