# Supplementary material for: The Effect of Folic Acid Supplementation on Endothelial Function and Arterial Stiffness Markers in Adults: A Systematic Review and Meta-Analysis
Source: Healthcare (Basel). 2023 Sep 13;11(18):2524. doi: 10.3390/healthcare11182524 (PMC10531078; doi:10.3390/healthcare11182524)

Figure S1

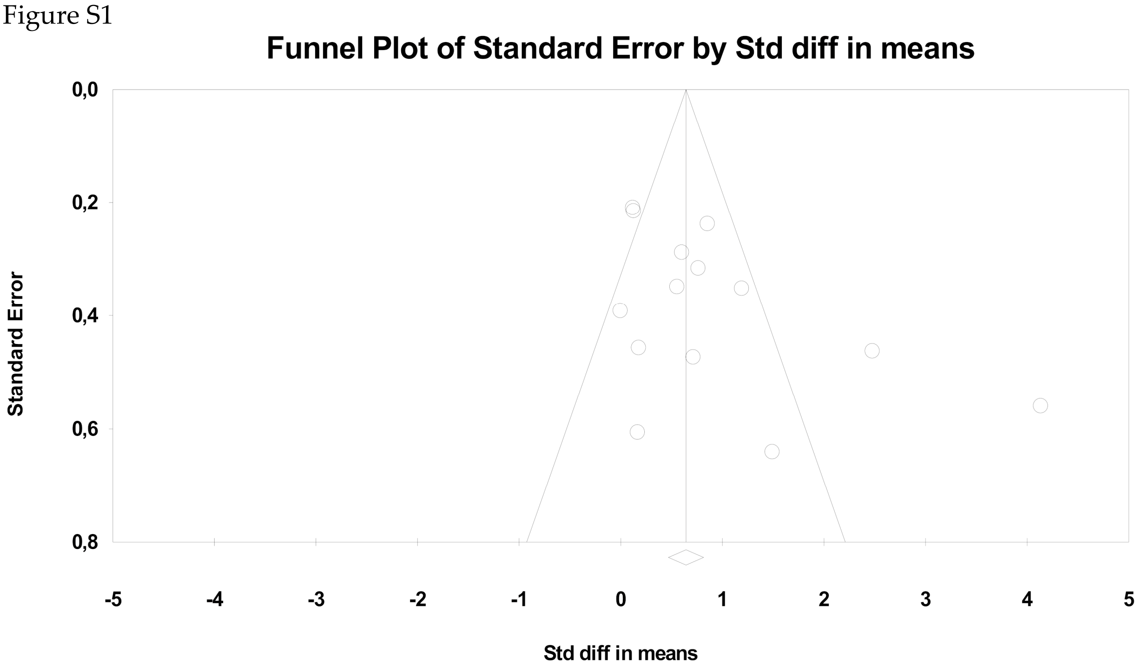

Figure S2

Study name	Time point	Statistics for each study							Sample size		Weight (Random)		
		Std diff in means	Standard error	Variance	Lower limit	Upper limit	Z-Value	p-Value	Intervention group	Control group	Relative weight	Relative weight	Std Residual
Grigoletti et al., 2018	4 weeks	0,170	0,607	0,368	-1,019	1,359	0,280	0,780	6	5	6,20		-0,75
Hoch et al., 2009	6 weeks	1,496	0,641	0,411	0,239	2,752	2,333	0,020	8	5	5,95		0,60
Moens et al., 2007	6 weeks	4,138	0,560	0,314	3,039	5,236	7,384	0,000	20	20	6,55		3,42
Shirodaria et al., 2007	7 weeks	0,767	0,317	0,100	0,145	1,388	2,418	0,016	42	14	8,42		-0,16
Yilmaz et al., 2007	8 weeks	1,195	0,353	0,124	0,504	1,886	3,388	0,001	20	18	8,16		0,35
Title et al., 2006	2 weeks	0,717	0,474	0,225	-0,212	1,646	1,513	0,130	9	10	7,22		-0,20
Lekakis et al., 2004	4 weeks	0,558	0,350	0,122	-0,127	1,243	1,596	0,110	17	17	8,18		-0,41
Woodman et al., 2004	8 weeks	0,000	0,392	0,154	-0,769	0,769	0,000	1,000	13	13	7,86		-1,05
Doshi et al., 2002	6 weeks	2,481	0,463	0,215	1,573	3,389	5,355	0,000	16	17	7,31		1,77
Miner et al., 2001	10 weeks	0,181	0,457	0,209	-0,716	1,077	0,395	0,693	12	8	7,35		-0,81
Thambyrajah et al., 2001	12 weeks	0,129	0,216	0,047	-0,294	0,553	0,600	0,549	43	43	9,08		-0,97
Thambyrajah et al., 2000	12 weeks	0,123	0,210	0,044	-0,289	0,534	0,584	0,559	47	44	9,11		-0,98
Title et al., 2000	17 weeks	0,605	0,289	0,084	0,038	1,172	2,092	0,036	25	25	8,62		-0,36
		0,899	0,251	0,063	0,408	1,391	3,588	0,000					

Std diff in means and 95% CI

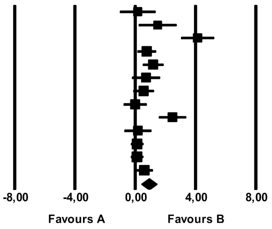

Figure S3

Study name	Time point	Statistics with study removed						
		Point	Standard error	Variance	Lower limit	Upper limit	Z-Value	p-Value
Grigoletti et al., 2018	4 weeks	0,932	0,235	0,055	0,472	1,392	3,971	0,000
Hoch et al., 2009	6 weeks	0,855	0,232	0,054	0,399	1,310	3,678	0,000
Moens et al., 2007	6 weeks	0,660	0,165	0,027	0,336	0,984	3,991	0,000
Shirodaria et al., 2007	7 weeks	0,904	0,245	0,060	0,424	1,384	3,695	0,000
Yilmaz et al., 2007	8 weeks	0,866	0,239	0,057	0,398	1,334	3,625	0,000
Moat et al., 2006	6 weeks	0,899	0,251	0,063	0,408	1,391	3,588	0,000
Title et al., 2006	2 weeks	0,903	0,238	0,057	0,436	1,370	3,788	0,000
Lekakis et al., 2004	4 weeks	0,920	0,243	0,059	0,444	1,396	3,786	0,000
Woodman et al., 2004	8 weeks	0,959	0,237	0,056	0,495	1,423	4,049	0,000
Doshi et al., 2002	6 weeks	0,763	0,213	0,045	0,347	1,180	3,589	0,000
Miner et al., 2001	10 weeks	0,941	0,237	0,056	0,476	1,406	3,966	0,000
Thambyrajah et al., 2001	12 weeks	0,963	0,244	0,060	0,484	1,442	3,940	0,000
Thambyrajah et al., 2000	12 weeks	0,964	0,244	0,060	0,485	1,442	3,947	0,000
Title et al., 2000	17 weeks	0,919	0,247	0,061	0,435	1,403	3,723	0,000
		0,888	0,225	0,051	0,447	1,329	3,944	0,000

Std diff in means (95% CI) with study removed

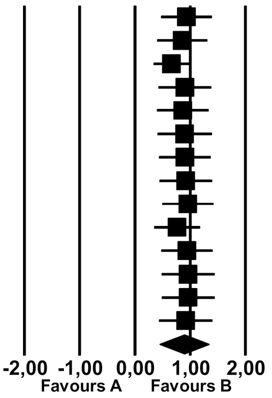

Figure S4

Study name	Time point	Cumulative statistics									Relative weight
		Point	Standard error	Variance	Lower limit	Upper limit	Z-Value	p-Value	Intervention group	Control group	
Thambyrajah et al., 2000	12 weeks	0,123	0,210	0,044	-0,289	0,534	0,584	0,559	47	44	8,54
Title et al., 2000	17 weeks	0,323	0,238	0,057	-0,143	0,789	1,358	0,174	72	69	16,54
Mner et al., 2001	10 weeks	0,276	0,159	0,025	-0,036	0,588	1,732	0,083	84	77	23,22
Thambyrajah et al., 2001	12 weeks	0,224	0,128	0,016	-0,027	0,476	1,750	0,080	127	120	31,72
Doshi et al., 2002	6 weeks	0,629	0,326	0,106	-0,010	1,268	1,928	0,054	143	137	38,36
Lekakis et al., 2004	4 weeks	0,607	0,274	0,075	0,070	1,144	2,214	0,027	160	154	45,90
Woodman et al., 2004	8 weeks	0,522	0,244	0,059	0,044	1,000	2,140	0,032	173	167	53,11
Moat et al., 2006	6 weeks	0,566	0,216	0,047	0,143	0,989	2,620	0,009	228	196	61,46
Title et al., 2006	2 weeks	0,576	0,200	0,040	0,185	0,967	2,884	0,004	237	206	68,01
Moens et al., 2007	6 weeks	0,895	0,287	0,082	0,333	1,457	3,120	0,002	257	226	73,88
Shirodaria et al., 2007	7 weeks	0,875	0,259	0,067	0,367	1,384	3,374	0,001	299	240	81,68
Yilmaz et al., 2007	8 weeks	0,899	0,243	0,059	0,423	1,375	3,704	0,000	319	258	89,20
Hoch et al., 2009	6 weeks	0,932	0,235	0,055	0,472	1,392	3,971	0,000	327	263	94,48
Grigoletti et al., 2018	4 weeks	0,888	0,225	0,051	0,447	1,329	3,944	0,000	333	268	100,00
		0,888	0,225	0,051	0,447	1,329	3,944	0,000			

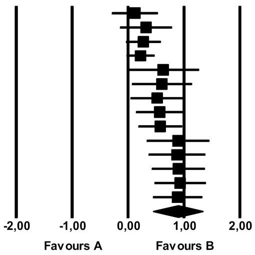

Figure S5

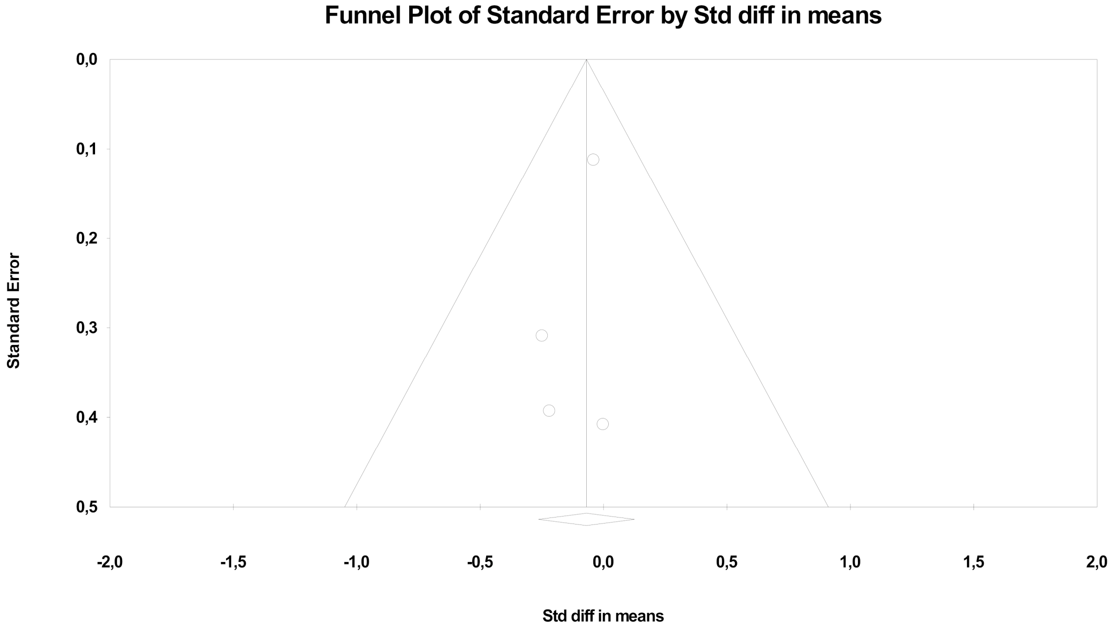

Figure S6

Study name	Time point	Statistics with study removed						
		Point	Standard error	Variance	Lower limit	Upper limit	Z-Value	p-Value
Shirodaria et al., 2007	7 weeks	-0,049	0,105	0,011	-0,254	0,156	-0,466	0,641
Zoungas et al., 2006	188 weeks	-0,174	0,209	0,044	-0,584	0,235	-0,833	0,405
Mangoni et al., 2005	4 weeks	-0,059	0,103	0,011	-0,260	0,142	-0,577	0,564
Mangoni et al., 2002	4 weeks	-0,074	0,102	0,010	-0,274	0,127	-0,719	0,472
		-0,069	0,099	0,010	-0,264	0,125	-0,698	0,485

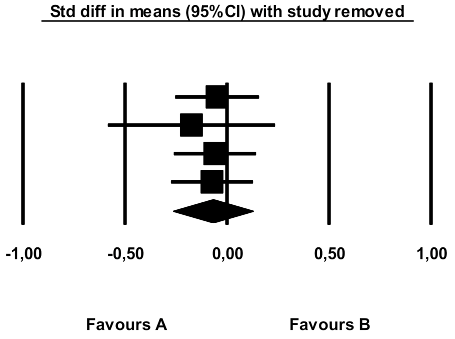

Figure S7

Study name	Time point	Cumulative statistics									Relative weight
		Standard error	Variance	Lower limit	Point	Upper limit	Z-Value	p-Value	Intervention group	Control group	
Mangoni et al., 2002	4 weeks	0,408	0,167	-0,800	0,000	0,800	0,000	1,000	12	12	5,90
Mangoni et al., 2005	4 weeks	0,283	0,080	-0,668	-0,113	0,442	-0,398	0,690	25	25	12,26
Zoungas et al., 2006	188 weeks	0,105	0,011	-0,254	-0,049	0,156	-0,466	0,641	181	184	89,73
Shirodaria et al., 2007	7 weeks	0,099	0,010	-0,264	-0,069	0,125	-0,698	0,485	223	198	100,00
		0,099	0,010	-0,264	-0,069	0,125	-0,698	0,485			

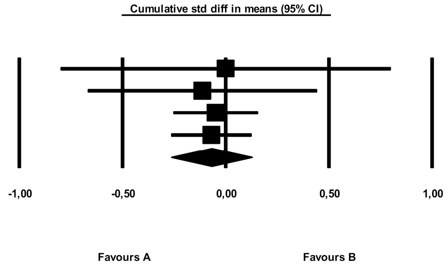

Figure S8

Study name	Time point	Point	Standard error	Variance	Lower limit	Upper limit	Z-Value	p-Value
		Statistics with study removed						
Mangoni et al, 2005	4 weeks	-0,114	0,089	0,008	-0,288	0,060	-1,284	0,199
Zoungas et al, 2004	206 weeks	0,325	0,395	0,156	-0,448	1,099	0,824	0,410
		-0,093	0,087	0,008	-0,263	0,077	-1,072	0,284

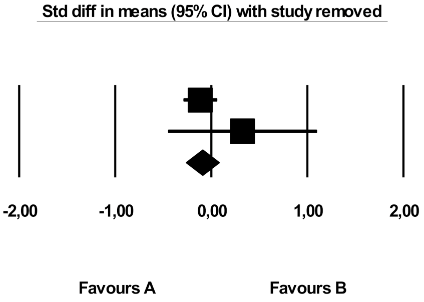

Figure S9

Study name	Time point	Point	Standard error	Variance	Lower limit	Upper limit	Z-Value	p-Value	Group-A	Group-B	Relative weight
		Cumulative statistics									
Zoungas et al, 2004	206 weeks	-0,114	0,089	0,008	-0,288	0,060	-1,284	0,199	315	212	95,17
Mangoni et al, 2005	4 weeks	-0,093	0,087	0,008	-0,263	0,077	-1,072	0,284	328	225	100,00
		-0,093	0,087	0,008	-0,263	0,077	-1,072	0,284			

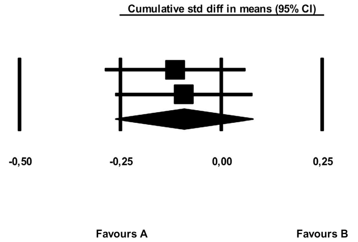

Figure S10

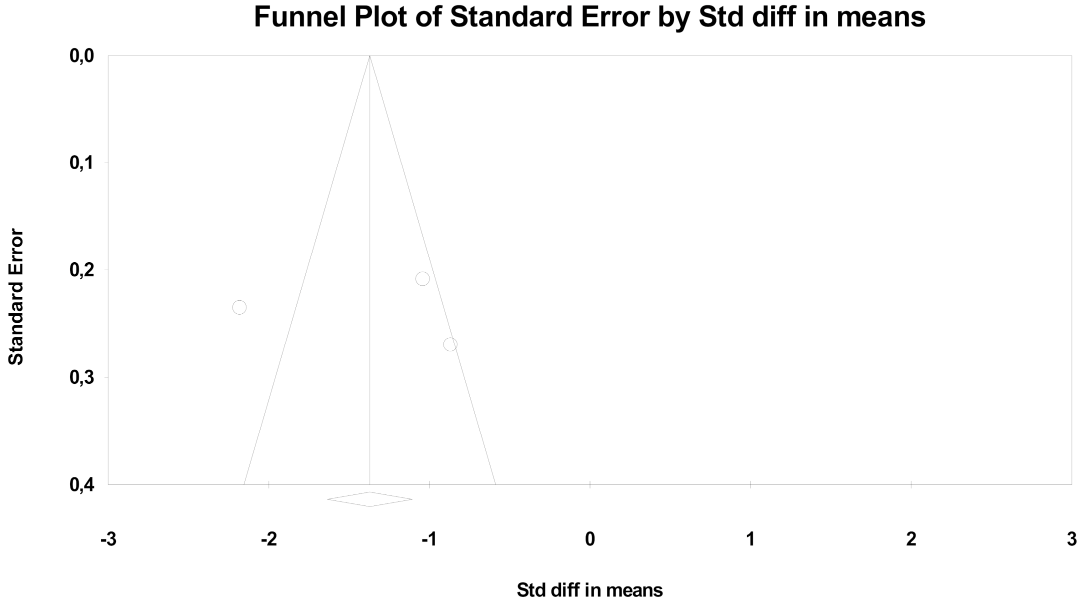

Figure S11

Study name	Time point	Point	Standard error	Variance	Lower limit	Upper limit	Z-Value	p-Value
		Statistics with study removed						
Li et al., 2020	26 weeks	-1,528	0,657	0,431	-2,815	-0,241	-2,327	0,020
Ma et al., 2019	24 weeks	-0,973	0,165	0,027	-1,297	-0,649	-5,890	0,000
Solini et al., 2006	12 weeks	-1,603	0,570	0,325	-2,721	-0,486	-2,812	0,005
		-1,364	0,408	0,167	-2,164	-0,563	-3,339	0,001

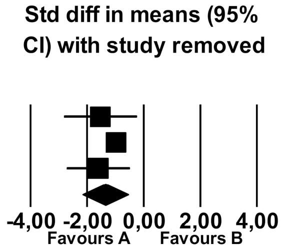

Figure S12

Study name	Time point	Cumulative statistics									Relative weight
		Point	Standard error	Variance	Lower limit	Upper limit	Z-Value	p-Value	Intervention group	Control group	
Solini et al., 2006	12 weeks	-0,865	0,270	0,073	-1,394	-0,336	-3,204	0,001	30	30	32,31
Ma et al., 2019	24 weeks	-1,528	0,657	0,431	-2,815	-0,241	-2,327	0,020	88	87	65,75
Li et al., 2020	26 weeks	-1,364	0,408	0,167	-2,164	-0,563	-3,339	0,001	141	138	100,00
		-1,364	0,408	0,167	-2,164	-0,563	-3,339	0,001			

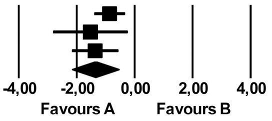

Figure S13

Group by: Duration of intervention	Study name	Time point	Std diff in means	Standard error	Variance	Lower limit	Upper limit	Z-Value	p-Value	Intervention group	Control group	Relative weight	Relative weight	Std Residual
			Statistics for each study							Sample size				
Long	Hoch et al., 2009	6 weeks	1,496	0,641	0,411	0,239	2,752	2,333	0,020	8	5	6,85		0,50
Long	Mbens et al., 2007	6 weeks	4,138	0,560	0,314	3,039	5,236	7,384	0,000	20	20	7,53		3,30
Long	Shirodaria et al., 2007	7 weeks	0,767	0,317	0,100	0,145	1,388	2,418	0,016	42	14	9,63		-0,28
Long	Yilmaz et al., 2007	8 weeks	1,195	0,353	0,124	0,504	1,886	3,388	0,001	20	18	9,34		0,23
Long	Mbat et al., 2006	6 weeks	0,858	0,239	0,057	0,389	1,326	3,591	0,000	55	29	10,21		-0,17
Long	Woodman et al., 2004	8 weeks	0,000	0,392	0,154	-0,769	0,769	0,000	1,000	13	13	9,00		-1,15
Long	Doshi et al., 2002	6 weeks	2,481	0,463	0,215	1,573	3,389	5,355	0,000	16	17	8,38		1,65
Long	Mner et al., 2001	10 weeks	0,181	0,457	0,209	-0,716	1,077	0,395	0,693	12	8	8,43		-0,91
Long	Thambyrajah et al., 2001	12 weeks	0,129	0,216	0,047	-0,294	0,553	0,600	0,549	43	43	10,36		-1,09
Long	Thambyrajah et al., 2000	12 weeks	0,123	0,210	0,044	-0,289	0,534	0,584	0,559	47	44	10,40		-1,10
Long	Title et al., 2000	17 weeks	0,605	0,289	0,084	0,038	1,172	2,092	0,036	25	25	9,85		-0,48
Long			0,996	0,272	0,074	0,464	1,528	3,668	0,000					
Short	Grigoletti et al., 2018	4 weeks	0,170	0,607	0,368	-1,019	1,359	0,280	0,780	6	5	17,70		-0,66
Short	Title et al., 2006	2 weeks	0,717	0,474	0,225	-0,212	1,646	1,513	0,130	9	10	29,00		0,46
Short	Lekakis et al., 2004	4 weeks	0,558	0,350	0,122	-0,127	1,243	1,596	0,110	17	17	53,30		0,09
Short			0,535	0,255	0,065	0,035	1,036	2,098	0,036					
Overall			0,752	0,186	0,035	0,387	1,116	4,041	0,000					

Std diff in means and 95% CI

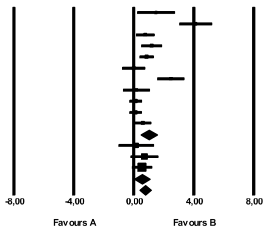

Figure S14

Group by: Age > 60 years	Study name	Time point	Std diff in means	Standard error	Variance	Lower limit	Upper limit	Z-Value	p-Value	Intervention group	Control group	Relative weight	Relative weight	Std Residual	I
			Statistics for each study							Sample size					
No	Grigoletti et al., 2018	4 weeks	0,170	0,607	0,368	-1,019	1,359	0,280	0,780	6	5	8,86		-0,86	
No	Hoch et al., 2009	6 weeks	1,496	0,641	0,411	0,239	2,752	2,333	0,020	8	5	8,58		0,33	
No	Mbens et al., 2007	6 weeks	4,138	0,560	0,314	3,039	5,236	7,384	0,000	20	20	9,23		2,79	
No	Yilmaz et al., 2007	8 weeks	1,195	0,353	0,124	0,504	1,886	3,388	0,001	20	18	10,81		0,08	
No	Title et al., 2006	2 weeks	0,717	0,474	0,225	-0,212	1,646	1,513	0,130	9	10	9,91		-0,39	
No	Lekakis et al., 2004	4 weeks	0,558	0,350	0,122	-0,127	1,243	1,596	0,110	17	17	10,83		-0,57	
No	Woodman et al., 2004	8 weeks	0,000	0,392	0,154	-0,769	0,769	0,000	1,000	13	13	10,53		-1,11	
No	Doshi et al., 2002	6 weeks	2,481	0,463	0,215	1,573	3,389	5,355	0,000	16	17	9,99		1,31	
No	Mner et al., 2001	10 weeks	0,181	0,457	0,209	-0,716	1,077	0,395	0,693	12	8	10,04		-0,91	
No	Title et al., 2000	17 weeks	0,605	0,289	0,084	0,038	1,172	2,092	0,036	25	25	11,22		-0,53	
No			1,120	0,345	0,119	0,443	1,797	3,242	0,001						
Yes	Shirodaria et al., 2007	7 weeks	0,767	0,317	0,100	0,145	1,388	2,418	0,016	42	14	19,90		0,81	
Yes	Mbat et al., 2006	6 weeks	0,858	0,239	0,057	0,389	1,326	3,591	0,000	55	29	25,35		1,22	
Yes	Thambyrajah et al., 2001	12 weeks	0,129	0,216	0,047	-0,294	0,553	0,600	0,549	43	43	27,14		-0,94	
Yes	Thambyrajah et al., 2000	12 weeks	0,123	0,210	0,044	-0,289	0,534	0,584	0,559	47	44	27,61		-0,97	
Yes			0,439	0,201	0,040	0,046	0,832	2,188	0,029						
Overall			0,611	0,173	0,030	0,271	0,951	3,520	0,000						

Std diff in means and 95% CI

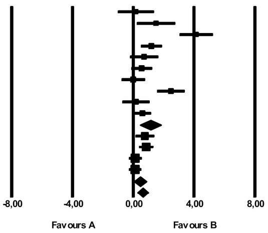

Figure S15

Group by: Country	Study name	Time point	Std diff in means	Standard error	Variance	Lower limit	Upper limit	Z-Value	p-Value	Intervention group	Control group	Relative weight	Relative weight	Std Residual
			Statistics for each study							Sample size				
Europe/North America/Australia	Hoch et al., 2009	6 weeks	1,496	0,641	0,411	0,239	2,752	2,333	0,020	8	5	6,19		0,60
Europe/North America/Australia	Mbens et al., 2007	6 weeks	4,138	0,560	0,314	3,039	5,236	7,384	0,000	20	20	6,85		3,50
Europe/North America/Australia	Shirodaria et al., 2007	7 weeks	0,767	0,317	0,100	0,145	1,388	2,418	0,016	42	14	8,93		-0,18
Europe/North America/Australia	Mbat et al., 2006	6 weeks	0,858	0,239	0,057	0,389	1,326	3,591	0,000	55	29	9,52		-0,07
Europe/North America/Australia	Title et al., 2006	2 weeks	0,717	0,474	0,225	-0,212	1,646	1,513	0,130	9	10	7,59		-0,22
Europe/North America/Australia	Lekakis et al., 2004	4 weeks	0,558	0,350	0,122	-0,127	1,243	1,596	0,110	17	17	8,66		-0,44
Europe/North America/Australia	Woodman et al., 2004	8 weeks	0,000	0,392	0,154	-0,769	0,769	0,000	1,000	13	13	8,30		-1,10
Europe/North America/Australia	Doshi et al., 2002	6 weeks	2,481	0,463	0,215	1,573	3,389	5,355	0,000	16	17	7,68		1,81
Europe/North America/Australia	Mner et al., 2001	10 weeks	0,181	0,457	0,209	-0,716	1,077	0,395	0,693	12	8	7,73		-0,85
Europe/North America/Australia	Thambyrajah et al., 2001	12 weeks	0,129	0,216	0,047	-0,294	0,553	0,600	0,549	43	43	9,68		-1,03
Europe/North America/Australia	Thambyrajah et al., 2000	12 weeks	0,123	0,210	0,044	-0,289	0,534	0,584	0,559	47	44	9,72		-1,04
Europe/North America/Australia	Title et al., 2000	17 weeks	0,605	0,289	0,084	0,038	1,172	2,092	0,036	25	25	9,15		-0,39
Europe/North America/Australia			0,913	0,250	0,063	0,423	1,403	3,651	0,000					
Other	Grigoletti et al., 2018	4 weeks	0,170	0,607	0,368	-1,019	1,359	0,280	0,780	6	5	38,41		-1,00
Other	Yilmaz et al., 2007	8 weeks	1,195	0,353	0,124	0,504	1,886	3,388	0,001	20	18	61,59		1,00
Other			0,801	0,499	0,249	-0,176	1,778	1,607	0,108					
Overall			0,890	0,224	0,050	0,452	1,329	3,984	0,000					

Std diff in means and 95% CI

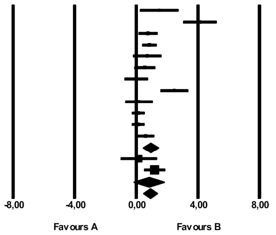

Figure S16

Group by: Fortification	Study name	Time point	Std diff in means	Standard error	Variance	Lower limit	Upper limit	Z-Value	p-Value	Intervention group	Control group	Relative weight	Relative weight	Std Residual
			Statistics for each study							Sample size				
No	Moens et al., 2007	6 weeks	4,138	0,560	0,314	3,039	5,236	7,384	0,000	20	20	9,11		3,15
No	Shirodaria et al., 2007	7 weeks	0,767	0,317	0,100	0,145	1,388	2,418	0,016	42	14	11,41		-0,32
No	Yilmaz et al., 2007	8 weeks	1,195	0,353	0,124	0,504	1,886	3,388	0,001	20	18	11,10		0,17
No	Moat et al., 2006	6 weeks	0,858	0,239	0,057	0,389	1,326	3,591	0,000	55	29	12,03		-0,22
No	Lekakis et al., 2004	4 weeks	0,558	0,350	0,122	-0,127	1,243	1,596	0,110	17	17	11,13		-0,55
No	Woodman et al., 2004	8 weeks	0,000	0,392	0,154	-0,769	0,769	0,000	1,000	13	13	10,74		-1,16
No	Doshi et al., 2002	6 weeks	2,481	0,463	0,215	1,573	3,389	5,355	0,000	16	17	10,06		1,55
No	Thambyrajah et al., 2001	12 weeks	0,129	0,216	0,047	-0,294	0,553	0,600	0,549	43	43	12,19		-1,10
No	Thambyrajah et al., 2000	12 weeks	0,123	0,210	0,044	-0,289	0,534	0,584	0,559	47	44	12,23		-1,11
No			1,043	0,311	0,097	0,434	1,652	3,356	0,001					
Yes	Grigoletti et al., 2018	4 weeks	0,170	0,607	0,368	-1,019	1,359	0,280	0,780	6	5	10,32		-0,72
Yes	Hoch et al., 2009	6 weeks	1,496	0,641	0,411	0,239	2,752	2,333	0,020	8	5	9,24		1,49
Yes	Title et al., 2006	2 weeks	0,717	0,474	0,225	-0,212	1,646	1,513	0,130	9	10	16,90		0,31
Yes	Miner et al., 2001	10 weeks	0,181	0,457	0,209	-0,716	1,077	0,395	0,693	12	8	18,16		-0,98
Yes	Title et al., 2000	17 weeks	0,605	0,289	0,084	0,038	1,172	2,092	0,036	25	25	45,39		0,10
Yes			0,584	0,195	0,038	0,202	0,966	2,999	0,003					
Overall			0,714	0,165	0,027	0,390	1,037	4,324	0,000					

Std diff in means and 95% CI

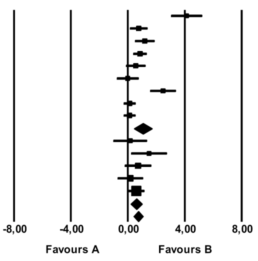

Figure S17

Group by<br>Duration ofintervention	Study name	Time point	Std diff in means	Standard error	Variance	Lower limit	Upper limit	Z-Value	p-Value	Intervention group	Control group	Relative weight	Relative weight	Std Residual
Long	Zoungas et al., 2006	188 weeks	-0,039	0,113	0,013	-0,260	0,182	-0,343	0,731	156	159	88,29		0,63
Long	Shirodaria et al., 2007	7 weeks	-0,247	0,309	0,096	-0,854	0,359	-0,799	0,424	42	14	11,71		-0,63
Long			-0,063	0,106	0,011	-0,271	0,144	-0,596	0,551					
Short	Mangoni et al., 2002	4 weeks	0,000	0,408	0,167	-0,800	0,800	0,000	1,000	12	12	48,15		0,38
Short	Mangoni et al., 2005	4 weeks	-0,218	0,393	0,155	-0,989	0,553	-0,553	0,580	13	13	51,85		-0,38
Short			-0,113	0,283	0,080	-0,668	0,442	-0,398	0,690					
Overall			-0,069	0,099	0,010	-0,264	0,125	-0,698	0,485					

(Statistics for each study: Std diff in means to p-Value; Sample size: Intervention group, Control group; Weight (Fixed): Relative weight, Relative weight, Std Residual)

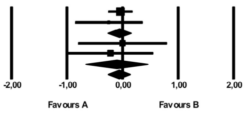

Figure S18

Group by<br>Age > 60 years	Study name	Time point	Std diff in means	Standard error	Variance	Lower limit	Upper limit	Z-Value	p-Value	Intervention group	Control group	Relative weight	Relative weight	Std Residual
No	Zoungas et al., 2006	188 weeks	-0,039	0,113	0,013	-0,260	0,182	-0,343	0,731	156	159	86,33		0,24
No	Mangoni et al., 2005	4 weeks	-0,218	0,393	0,155	-0,989	0,553	-0,553	0,580	13	13	7,09		-0,45
No	Mangoni et al., 2002	4 weeks	0,000	0,408	0,167	-0,800	0,800	0,000	1,000	12	12	6,58		0,12
No			-0,049	0,105	0,011	-0,254	0,156	-0,466	0,641					
Yes	Shirodaria et al., 2007	7 weeks	-0,247	0,309	0,096	-0,854	0,359	-0,799	0,424	42	14	100,00		-0,61
Yes			-0,247	0,309	0,096	-0,854	0,359	-0,799	0,424					
Overall			-0,069	0,099	0,010	-0,264	0,125	-0,698	0,485					

(Statistics for each study: Std diff in means to p-Value; Sample size: Intervention group, Control group; Weight (Fixed): Relative weight, Relative weight, Std Residual)

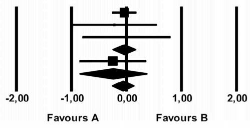

Figure S19

Group by<br>Duration of interventon	Study name	Time point	Std diff in means	Standard error	Variance	Lower limit	Upper limit	Z-Value	p-Value	Group-A	Group-B	Relative weight	Relative weight	Std Residual
Long	Zoungas et al, 2004	206 weeks	-0,114	0,089	0,008	-0,288	0,060	-1,284	0,199	315	212	100,00		-1,09
Long			-0,114	0,089	0,008	-0,288	0,060	-1,284	0,199					
Short	Mangoni et al, 2005	4 weeks	0,325	0,395	0,156	-0,448	1,099	0,824	0,410	13	13	100,00		1,09
Short			0,325	0,395	0,156	-0,448	1,099	0,824	0,410					
Overall			-0,093	0,087	0,008	-0,263	0,077	-1,072	0,284					

(Statistics for each study: Std diff in means to p-Value; Sample size: Group-A, Group-B; Weight (Fixed): Relative weight, Relative weight, Std Residual)

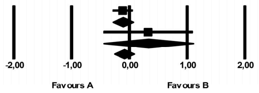

Figure S20

Group by<br>Age > 60 years	Study name	Time point	Std diff in means	Standard error	Variance	Lower limit	Upper limit	Z-Value	p-Value	Intervention group	Control group	Relative weight	Relative weight	Std Residual
No	Solini et al., 2006	12 weeks	-0,865	0,270	0,073	-1,394	-0,336	-3,204	0,001	30	30	100,00		
No			-0,865	0,270	0,073	-1,394	-0,336	-3,204	0,001					
Yes	Li et al., 2020	26 weeks	-1,038	0,209	0,044	-1,448	-0,629	-4,968	0,000	53	51	50,45		1,00
Yes	Ma et al., 2019	24 weeks	-2,178	0,235	0,055	-2,640	-1,717	-9,254	0,000	58	57	49,55		-1,00
Yes			-1,603	0,570	0,325	-2,721	-0,486	-2,812	0,005					
Overall			-1,000	0,244	0,060	-1,479	-0,522	-4,099	0,000					

(Statistics for each study: Std diff in means to p-Value; Sample size: Intervention group, Control group)

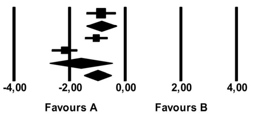

Figure S21

Group by<br>Country	Study name	Time point	Std diff in means	Standard error	Variance	Lower limit	Upper limit	Z-Value	p-Value	Intervention group	Control group	Relative weight	Relative weight	Std Residual
Europe/Noth America/Australia	Solini et al., 2006	12 weeks	-0,865	0,270	0,073	-1,394	-0,336	-3,204	0,001	30	30	100,00		
Europe/Noth America/Australia			-0,865	0,270	0,073	-1,394	-0,336	-3,204	0,001					
Other	Li et al., 2020	26 weeks	-1,038	0,209	0,044	-1,448	-0,629	-4,968	0,000	53	51	50,45		1,00
Other	Ma et al., 2019	24 weeks	-2,178	0,235	0,055	-2,640	-1,717	-9,254	0,000	58	57	49,55		-1,00
Other			-1,603	0,570	0,325	-2,721	-0,486	-2,812	0,005					
Overall			-1,000	0,244	0,060	-1,479	-0,522	-4,099	0,000					

(Statistics for each study: Std diff in means to p-Value; Sample size: Intervention group, Control group)

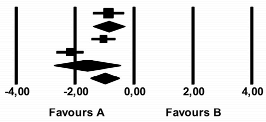

Supplement: Supplementary file 1 [file healthcare-11-02524-s001.zip › figures.pdf]
